# Effects of Tai Chi-based combined interventions on muscle function, muscle mass, and physical performance in older adults with sarcopenia, frailty, or risk of functional decline: a systematic review and meta-analysis

**DOI:** 10.3389/fpubh.2026.1891703

**Published:** 2026-08-20

**Authors:** Xudong Jin, Enliang Hu, Caiduo Shi

**Affiliations:** 1 School of Physical Education, Yunnan Minzu University, Kunming, China; 2 College of Aviation and Sports, Civil Aviation Flight University of China, Deyang, China

**Keywords:** combined intervention, frailty, functional decline, meta-analysis, older adults, sarcopenia, Tai Chi

## Abstract

**Background and objective:**

Sarcopenia and frailty involve declines in muscle strength, muscle mass, and physical performance, and single Tai Chi may be insufficient to cover all functional needs. Tai Chi-based combined interventions have emerged, but their overall effects remain unclear. This study evaluated their effects in older adults with sarcopenia, frailty, or risk of functional decline.

**Methods:**

We systematically searched PubMed, Embase, CENTRAL, Web of Science, Scopus, CINAHL, ClinicalTrials.gov, CNKI, Wanfang, and VIP from database inception to May 12, 2026. Randomized controlled trials and cluster-randomized controlled trials comparing Tai Chi-based combined interventions with non-exercise, active-exercise, or single Tai Chi controls were included. Risk-domain composite standardized mean differences (SMDs) were used for the primary analysis, assuming rho = 0.5. A restricted maximum-likelihood random-effects model with Hartung–Knapp adjustment was applied. Risk of bias was assessed using the Cochrane Risk of Bias 2 tool, and certainty of evidence was assessed using the Grading of Recommendations Assessment, Development and Evaluation approach.

**Results:**

A total of 21 studies (22 reports) were included, of which 16 contributed to quantitative synthesis. In the core layer, compared with active exercise control, combined interventions showed a potentially favorable estimate in the frailty/overall physical performance domain (SMD = 0.63, 95% CI: 0.05–1.21), whereas muscle function and muscle mass/quality estimates remained uncertain. Compared with non-exercise control, the muscle function domain (SMD = 1.00, 95% CI: 0.12–1.88) and frailty/overall physical performance domain (SMD = 1.35, 95% CI: 0.38–2.31) showed favorable estimates, whereas muscle mass/quality did not improve consistently. Among 14 GRADE bodies of evidence, 2 were Low and 12 Very low, with none Moderate or High.

**Conclusion:**

Tai Chi-based combined interventions may improve selected aspects of muscle function and overall physical performance, but the evidence was based on few heterogeneous studies and was of Low or Very low certainty. Additional benefits over active exercise or single Tai Chi, as well as the optimal intervention combination and dose, remain uncertain.

**Systematic review registration:**

The systematic review was registered in PROSPERO (CRD420261387528) https://www.crd.york.ac.uk/PROSPERO/view/CRD420261387528.

## 1 Introduction

Population aging has made sarcopenia, frailty, and functional decline important public health issues in older adult health management (1, 2). The global population aged 60 years and older is projected to increase from approximately 1 billion in 2020 to 1.4 billion in 2030 and 2.1 billion in 2050 (1). As the older population expands, the demand for health services related to reduced muscle strength, impaired physical performance, falls, disability, and long-term care will continue to increase (1, 2).

Sarcopenia, frailty, and functional decline have overlapping functional phenotypes involving declines in muscle strength, muscle mass, gait, balance, and physical performance (2, 3). The European Working Group on Sarcopenia in Older People 2 (EWGSOP2) identifies low muscle strength as the core characteristic of sarcopenia, confirms the diagnosis by reduced muscle quantity or quality, and uses reduced physical performance to indicate severity (3). Frailty refers to reduced physiological reserve and increased vulnerability to stressors, whereas functional decline refers to worsening mobility or ability to perform daily activities independently (2). These conditions increase falls, fractures, disability, reduced quality of life, and care burden (2, 4, 5), supporting early intervention for older adults at risk (1, 6).

Tai Chi has low equipment dependence, gentle movements, adjustable intensity, and relatively high community acceptability, making it suitable for implementation among older adults (7). Its training process includes elements such as weight shifting, lower-limb support, postural control, coordination, and attentional control. Accordingly, existing studies have mainly focused on its effects on balance, gait control, and fall prevention (8).

However, sarcopenia and frailty involve more than impaired balance or coordination and include reduced strength, muscle mass, endurance, physical performance, and daily function (2, 3, 6). Single Tai Chi may emphasize postural control and balance, with limited coverage of muscle mass gain, maximal strength, and higher-load stimuli (9). Combining Tai Chi with resistance/strength, endurance, balance, functional training, or physical activity promotion may therefore better address these needs (6, 10). The World Health Organization (WHO) physical activity guidelines and the International Exercise Recommendations in Older Adults (ICFSR) expert consensus guidelines emphasize functional balance, strength, and multicomponent physical activity for older adults (6, 10).

Existing systematic reviews have mainly evaluated the effects of single Tai Chi on falls, balance, mobility, and physical performance in older adults (8, 9). Relevant evidence suggests that Tai Chi may help improve balance and fall-risk-related outcomes (8, 9), but these studies mainly answer whether single Tai Chi is effective and cannot directly infer whether Tai Chi, as a component of combined interventions, has additional or more comprehensive functional benefits.

In recent years, some RCTs have combined Tai Chi with resistance, endurance, balance, functional training, or physical activity promotion. Findings from these trials were heterogeneous: some reported improvements in grip strength, sit-to-stand performance, gait, balance, or overall physical performance, whereas others found little additional benefit over active exercise or single Tai Chi, and muscle mass/quality findings were inconsistent (11–21). Existing reviews have not sufficiently synthesized “interventions containing a Tai Chi component” as an independent model or stratified estimates by comparator and target population. Therefore, this evidence requires reintegration from the perspectives of intervention structure, comparison type, target population, and GRADE certainty.

This study aimed to systematically evaluate the effects of Tai Chi-based combined interventions on muscle function, muscle mass/quality, overall physical performance, and balance/mobility in older adults with sarcopenia, frailty, or risk of functional decline. Comparisons were stratified by non-exercise control, active exercise control, and single Tai Chi control. The risk-domain composite SMD was used as the primary analysis, and key specific outcomes, sensitivity analyses, and GRADE certainty-of-evidence assessment were integrated to clarify the potential effects, robustness, and evidence boundaries of this type of combined intervention.

## 2 Methods

### 2.1 Study design, registration, and reporting guideline

This systematic review and meta-analysis was reported in accordance with the PRISMA 2020 guideline (22) and registered in PROSPERO (CRD420261387528) (23). The review question, eligibility criteria, and study-selection procedures followed the prespecified PICOS framework (24). Before formal quantitative synthesis, the primary analytical level was clarified as risk-domain composite standardized mean differences, while specific-outcome pools were retained as sensitivity or supplementary analyses to reduce repeated weighting of multiple correlated outcomes reported within the same study.

### 2.2 Literature search and study selection

We systematically searched PubMed, Embase, CENTRAL, Web of Science, Scopus, CINAHL, ClinicalTrials.gov, CNKI, Wanfang, and VIP from inception to May 12, 2026. The search strategy was constructed around Tai Chi and its synonyms, combined or multicomponent exercise interventions, older adults with functional decline or sarcopenia/frailty-related conditions, and randomized controlled trials. English-language databases were searched using a combination of subject headings and free-text terms, and Chinese-language databases were searched using combinations of keywords such as Tai Chi, older adults, sarcopenia, frailty, falls, physical function, and randomized trials. The full search strategies are provided in Supplementary Table S2.

After duplicate records were removed, titles and abstracts were screened, followed by full-text assessment of potentially eligible articles. Study selection was independently performed by two reviewers according to the prespecified PICOS criteria. Disagreements were resolved through discussion; if inconsistency remained, a third reviewer made the final decision.

### 2.3 Inclusion and exclusion criteria

The inclusion criteria were defined according to the PICOS framework. P: population eligibility was assessed at the study level. The standard route required an original trial-defined minimum age of 60 or 65 years and sarcopenia, frailty, prefrailty, fall risk, reduced physical function, or another explicit risk of functional decline; older populations without an explicit diagnostic or functional-risk condition were classified as extended-layer evidence. Mixed-age trials were retained as age-boundary evidence only when participants had an explicit functional-risk condition, all study-arm mean ages exceeded 60 years, and age-stratified outcomes were unavailable; their influence was examined in sensitivity analysis. I: the intervention combined Tai Chi or a Tai Chi variant with at least one other exercise or physical activity promotion component. C: controls included non-exercise, active exercise, and single Tai Chi. O: outcomes included muscle function, muscle mass/quality, overall physical performance, balance/mobility, and fall-related outcomes. S: RCTs and cRCTs were included.

Exclusion criteria included non-randomized studies, observational studies, case reports, reviews, conference abstracts, or studies without sufficient data; studies in which the intervention did not contain a Tai Chi component, involved only single Tai Chi, or could not separate the effects of Tai Chi from other co-interventions; studies in which the population did not fall within the scope of older adults or risk of functional decline; and studies in which outcomes were unrelated to the prespecified muscle function, physical performance, or balance/mobility outcomes.

### 2.4 Data extraction, intervention coding, and outcome classification

Data extraction was independently completed using prespecified forms and cross-checked. Extracted information included study design, study setting, participants’ functional status, diagnostic or screening criteria, sample size, intervention components, control type, intervention frequency, session duration, total intervention period, supervision mode, adherence, adverse events, outcome measures, measurement time points, means, standard deviations, change values, number of events, and other data available for effect size calculation. For multiarm studies, separable intervention and control arms were extracted according to the prespecified comparison types to avoid repeated counting of the same sample in the same pooled model (25, 26).

Interventions containing a Tai Chi component were coded by their main training components: Tai Chi plus resistance or strength training (TC + RT/ST), resistance/strength plus endurance training (TC + RT/ST + ET), balance training (TC + BT), functional training (TC + FT), multicomponent exercise (TC + MCE), and Tai Chi snacking plus physical activity promotion (TC-snack+PE). Control conditions were classified as non-exercise control, active exercise control, and single Tai Chi control. Full study characteristics, intervention components, and control types are provided in Supplementary Table S3.

Original outcomes were first standardized into specific outcome pools and then grouped into four risk domains according to their clinical meaning: the sarcopenia-related muscle function domain, sarcopenia-related muscle mass/quality domain, frailty/overall physical performance domain, and fall-risk/balance-mobility domain. Specific outcome pools were used for description and sensitivity analyses, whereas risk-domain outcomes were used for the subsequent primary analysis of composite SMDs. The full outcome mapping is provided in Supplementary Table S3.

### 2.5 Effect size calculation and statistical analysis

Continuous outcomes were expressed as standardized mean differences (SMDs), using Hedges’ g to correct for small-sample bias (27). Effect directions were harmonized so that positive values favored Tai Chi-based combined interventions. For lower-is-better indicators, including the Timed Up and Go test (TUG), five-times sit-to-stand test (5TCS), stride time, and walking completion time, the direction was reversed before calculation. Grip strength, lower-limb muscle strength, the Short Physical Performance Battery (SPPB), gait speed, six-minute walk test (6MWT), balance maintenance time, and muscle mass/quality were treated as higher-is-better outcomes.

The primary analysis used risk-domain composite SMDs. For multiple related indicators reported within the same study, comparison type, data model, and risk domain, these indicators were first combined at the study level into a study-level composite SMD (26), which was then entered into the overall synthesis. The within-study synthesis of multiple indicators used an equal-weighting method and accounted for correlations between indicators. Rho = 0.5 was set as the primary-analysis assumption, and rho = 0.3 and rho = 0.7 were used for sensitivity analyses (26). If a study provided only one indicator within a given risk domain, that indicator was directly used as the risk-domain effect size for that study. Specific outcome-pool analyses were used as sensitivity or supplementary analyses.

For cluster-randomized controlled trials (cRCTs), cluster-adjusted estimates or available standard errors reported in the original articles were prioritized. If only unadjusted group means, standard deviations, and sample sizes were reported, the effective sample size (ESS) was calculated from the intracluster correlation coefficient (ICC) and design effect and used for SMD variance estimation (28). For continuous outcomes, ESS correction retained the original means and standard deviations and adjusted only the sample size (28). The cRCT correction procedures and ICC assumptions are shown in Supplementary Table S9.

Statistical analyses were performed in the R environment. The primary analysis used a random-effects model, with τ^2^ estimated using restricted maximum likelihood (REML) (29), and pooled effects and 95% confidence intervals (CIs) were calculated using the Hartung–Knapp adjustment (30). I^2^, τ^2^ (31), and 95% prediction intervals (32) were also reported. Prediction intervals were used as supplementary information on heterogeneity and extrapolation because their interpretation is less stable when few studies are available. Quantitative synthesis was performed only when the same meta-analysis pool included at least 2 independent studies; results with k < 2 were reported narratively. Sensitivity analyses included rho assumptions, core versus core+extended layers, fixed-effect models, leave-one-out analysis, specific outcome pools, generalized inverse-variance (GIV) supplementary analysis, and event data. An additional sensitivity analysis excluded the age-boundary Sharma trial from the active-exercise-control, post-intervention, core-layer frailty/overall physical performance composite, using the same REML and Hartung–Knapp model; the TUG pool was not synthesized if only one study remained. Formal subgroup analysis or meta-regression by intervention component, frequency, session duration, or intervention period was not performed because individual pools contained only 2–4 studies. Publication bias and small-study effects were assessed only when k ≥ 10 (33), using funnel plots, Egger’s test (34), and trim-and-fill (35).

### 2.6 Risk-of-bias and certainty-of-evidence assessment

Risk of bias was assessed using the Cochrane Risk of Bias 2 (RoB 2) tool (36). Individually randomized controlled trials (RCTs) were assessed using the standard RoB 2 tool, whereas cluster-randomized controlled trials (cRCTs) were assessed using the cluster-randomized version. Domains included the randomization process, deviations from intended interventions, missing outcome data, outcome measurement, selection of the reported result, and cRCT-specific domains. Judgments were made at the outcome level, and overall risk was classified as Low, Some concerns, or High. Two reviewers completed assessments independently, with disagreements resolved through discussion or third-reviewer adjudication.

Certainty of evidence was assessed using the Grading of Recommendations Assessment, Development and Evaluation (GRADE) approach (37). Downgrading domains included risk of bias, inconsistency, indirectness, imprecision, and publication bias. Final certainty was classified as High, Moderate, Low, or Very low (38). GRADE was applied at two levels: risk-domain composite SMDs and key specific outcomes. Supplementary Table S4 presents the effect estimate, number of studies, heterogeneity, certainty, and main reasons for downgrading for each body of evidence.

This study further conducted a supplementary GRADE robustness audit. The RoB 2 audit assessed whether each body of evidence still had at least two independent studies available for quantitative synthesis after excluding studies with overall High RoB. The imprecision audit compared GRADE judgments under different 95% CI threshold rules. The related audit results are provided in Supplementary Table S8.

## 3 Results

### 3.1 Study selection process

**Figure 1:**
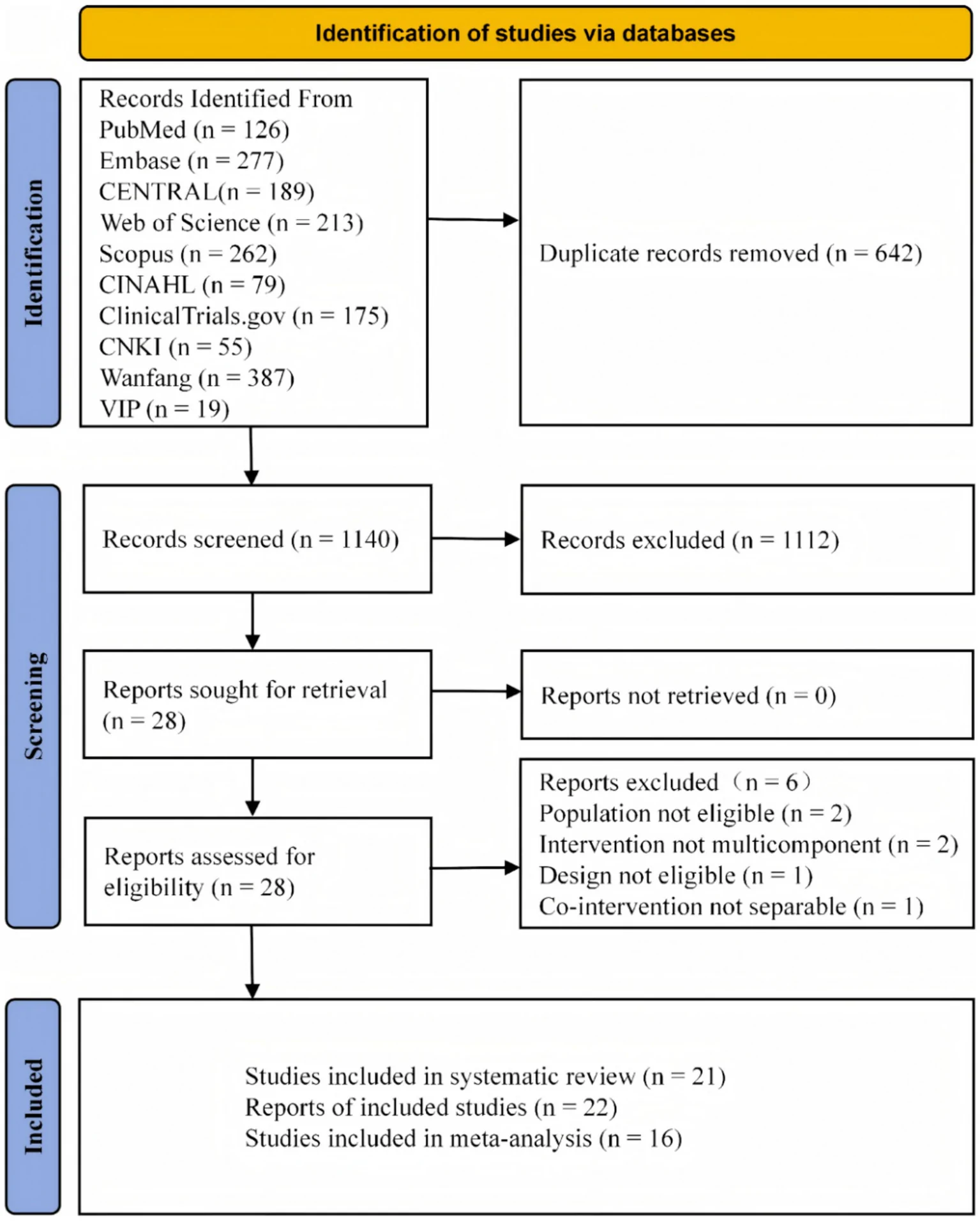
PRISMA flow diagram of study selection.

The search yielded 1,782 records. After 642 duplicate records were removed, 1,140 records underwent title and abstract screening, of which 1,112 were excluded. Twenty-eight reports were sought and retrieved for full-text assessment. Six reports were excluded because of an ineligible population (*n* = 2), an intervention that was not multicomponent (*n* = 2), an ineligible design (*n* = 1), or a non-separable co-intervention (*n* = 1). Finally, 21 independent studies represented by 22 reports were included in the systematic review, and 16 studies contributed to at least one quantitative synthesis. The study-selection process is shown in Figure 1.

### 3.2 Characteristics of included studies and intervention combinations

A total of 21 independent studies represented by 22 reports were included. Individual study sample sizes ranged from 18 (39) to 914 (40), and publication years ranged from 1993 (11) to 2026 (41, 42). Studies were conducted in China, the United States, the United Kingdom, India, Finland, and Peru (11, 12, 40, 43–46). Eighteen studies were individually randomized RCTs and 3 were cRCTs (13, 14, 47). Of the 22 reports, 3 were published in Chinese and 19 in English; at the independent-study level, 2 studies were represented by Chinese-language reports and 19 by English-language reports because the two reports by Ma et al. originated from the same randomized sample. Reported baseline or group mean ages ranged from approximately 62.0 to 84.38 years. Nine studies were classified as core-layer populations and 12 as extended-layer populations. The basic characteristics are shown in Table 1. One age-boundary trial enrolled women aged 55–70 years. The original report described the recruited population as having a “history of risk of fall,” but did not report the number of participants with actual previous falls, individual fall-risk classifications, the number aged 55–59 years, or age-stratified outcomes. Although all study-arm mean ages exceeded 60 years, group-level baseline measures of balance, mobility, and concern about falling supported classification of the sample as risk-enriched age-boundary evidence rather than as a study meeting the standard minimum-age route (12).

**Table 1:** Summary characteristics of the included studies.

Study	Country	Design/level	Population	Sample size, *n* (R/A)	Age, y	Female, %	Intervention vs comparator	Outcomes
Ma (2020/2022) (56)	China	RCT/extended	General OA	TC + ST 50/NR; TC 50/NR; ST 47/NR; NEC 45/NR	66.69–68.62	NR	TC + ST vs. TC/ST/NEC	③④⑤⑧
Zhang (2021) (20)	China	RCT/extended	General OA; regular TC	Cmb 30/28; TC 30/28	66.6–67.4	39.3–42.9	TC + RT + ET vs. TC	①③
Judge (1993) (11)	USA	RCT/extended	Healthy older women	38/21 overall	67.8–68.5	100	TC + MCE vs. FLEX	④
Wolfson (1996) (43)	USA	Factorial RCT/extended	General OA ≥ 75 y	EDU 27/NR; BT 28/NR; ST 28/NR; BT + ST 27/NR	78.9–80.6	42 overall	BT/ST plus TC maintenance vs. EDU	①③④
Zhang (2022) (39)	China	Pilot RCT/extended	General OA	INT 9/NR; CON 9/NR	63 overall	55.6 overall	TC + BT vs. AEC	③④⑦
Chen (2023) (15)	China	RCT/core	Sarcopenia	INT 30/25; CON 30/24	65.21–65.68	100	TC + RT vs. NEC	①②③
Sharma (2025) (12)	India	3-arm RCT/core; age-boundary	Women with fall-related functional vulnerability	OEP 15/10; OEP + TC 15/10; WALK 15/10	62.0–64.4	100	OEP + TC vs. OEP/WALK	③④⑤
Meng (2022) (16)	China	3-arm RCT/core	Frailty	TC NR/50; Cmb NR/50; RT + ET NR/50	75.60–76.59	50.0–60.0	TC + RT + ET vs. TC/RT + ET	①③⑥
Rikkonen (2023) (40)	Finland	RCT/extended	Community older women	INT 457/NR; CON 457/NR	76.4–76.6	100	RT + TC vs. NEC	⑤
Woo (2024) (13)	China	cRCT/core	Prefrailty; men	INT 21/16; CON 22/18	84.31–84.38	0	TC + RT vs. EDU	①⑥⑧
Zhan (2026) (41)	China	3-arm RCT/core	Sarcopenia	HEG 16/16; EBG 17/17; CG 16/16	70.31–71.75	56.3–62.5	TC + RT vs. RT/EDU	①②③⑦⑧
Su (2015) (19)	China	3-arm RCT/extended	General OA	NEC 16/NR; TC 17/NR; WTC 16/NR	65.3 overall	41.2–50.0	WTC vs. TC/NEC	①
Alegre-Tamariz (2025) (44)	Peru	RCT/extended	Functionally independent OA	INT 29/NR; AEC 18/NR	72.66 overall	95.7 overall	PAW+TC + FGT vs. PAW	①③⑧
Liang (2022) (45)	UK	4-arm RCT/extended	Non-frail OA	ES 15/NR; TCS 16/NR; ES + TCS 15/NR; CON 17/NR	71.1–73.3	54 overall	ES + TCS vs. ES/TCS/CON	③④
Yang (2025) (21)	China	3-arm RCT/extended	Independent community OA	TC + RB 45/45; basic TC 45/45; 24-form TC 45/45	66.87–67.98	66.7–68.9	TC + RB vs. TC	①③④⑤⑦
Guo (2024) (17)	China	3-arm RCT/core	Sarcopenia	TC + ST NR/33; ST NR/30; EDU NR/30	65.42–66.94	46.7–57.6	TC + ST vs. ST/EDU	①②
Zhuang (2014) (18)	China	RCT/extended	Community OA	INT 28/22; NEC 28/28	65.4–66.3	68.2–75.0	TC + MCE vs. NEC	①③④
Liu (2022) (14)	China	cRCT/core	Prefrailty/frailty	INT 72/67; UC 71/68	80.74–80.75	70.37 overall	TC + MCE vs. UC	③⑥
Lin (2015) (47)	China	cRCT/extended	Community/day-care OA	INT 54/48; UC 54/47	73.9–74.2	62.5–66.0	TC + RT vs. UC	①③
Wei (2026) (42)	China	3-arm RCT/core	Sarcopenia	Hybrid NR/31; RT NR/30; EDU NR/30	65.42–66.87	46.7–56.7	Hybrid TC/RT vs. RT/EDU	①②⑥
Liang (2024) (46)	UK	RCT/core	Prefrailty/functional risk	INT 44/NR; WL 46/NR	74.1 overall	70.5–71.7	SS + TCS vs. WL	①③④

Age and female proportion are presented as the overall value or range across study arms. For multiarm studies, group data follow the order shown in the intervention and comparator column. Detailed eligibility criteria, group-level participant data, intervention dose, and exact outcome instruments are provided in Supplementary Table S3. AEC, active exercise control; BT, balance training; CG, control group; Cmb, combined intervention; CON, control; cRCT, cluster-randomized controlled trial; EBG, elastic-band group; EDU, education control; ES, exercise snacking; ET, endurance training; FGT, functional gait training; FLEX, flexibility exercise; HEG, hybrid exercise group; INT, intervention; MCE, multicomponent exercise; NEC, non-exercise control; NR, not reported; OA, older adults; OEP, Otago Exercise Programme; PAW, physical-activity workshop; R/A, randomized/analyzed sample size; RB, Roliball; RCT, randomized controlled trial; RT, resistance training; SS, strength snacking; ST, strength training; TC, Tai Chi; TCS, Tai Chi snacking; UC, usual care; WALK, walking control; WL, wait-list control; WTC, weighted Tai Chi. ①, muscle strength; ②, muscle mass or quality; ③, physical performance or mobility; ④, balance or postural control; ⑤, falls, fall risk, or fear of falling; ⑥, frailty; ⑦, cognitive function; and ⑧, activities of daily living, physical activity, quality of life, or other physiological or structural outcomes.

All interventions were based on Tai Chi or Tai Chi-derived forms combined with one or more exercise or functional training components (11, 18, 47). The main categories included Tai Chi combined with resistance or strength training, resistance/strength plus endurance training, balance training, functional gait training, multicomponent exercise, or physical activity promotion (11–14, 16, 18–20, 39, 44–47, 56). Intervention duration ranged from 4 weeks (39, 45) to 52 weeks (14). Most conventional programs were delivered 2–5 times per week, with reported session durations ranging from approximately 20 to 90 min, whereas two exercise-snacking programs used brief daily formats of approximately 10 min per session or 20 min per day. The relative contribution, intensity, progression, supervision, and adherence of the Tai Chi and accompanying components were not consistently reported.

Control conditions were classified as non-exercise control (NEC), active exercise control (AEC), and single Tai Chi control (STC). NEC mainly included health education, wait-list control, maintenance of daily living, or usual activities (14, 15, 18, 40). AEC included strength or resistance training, Otago exercise, flexibility training, usual physical activity, or other structured exercise (11, 12, 39, 44). STC comprised single Tai Chi or Tai Chi snacking (20, 21, 46). Six studies used single Tai Chi or Tai Chi-derived forms as a control, including one study with two Tai Chi control arms.

### 3.3 Risk-of-bias assessment results

**Figure 2:**
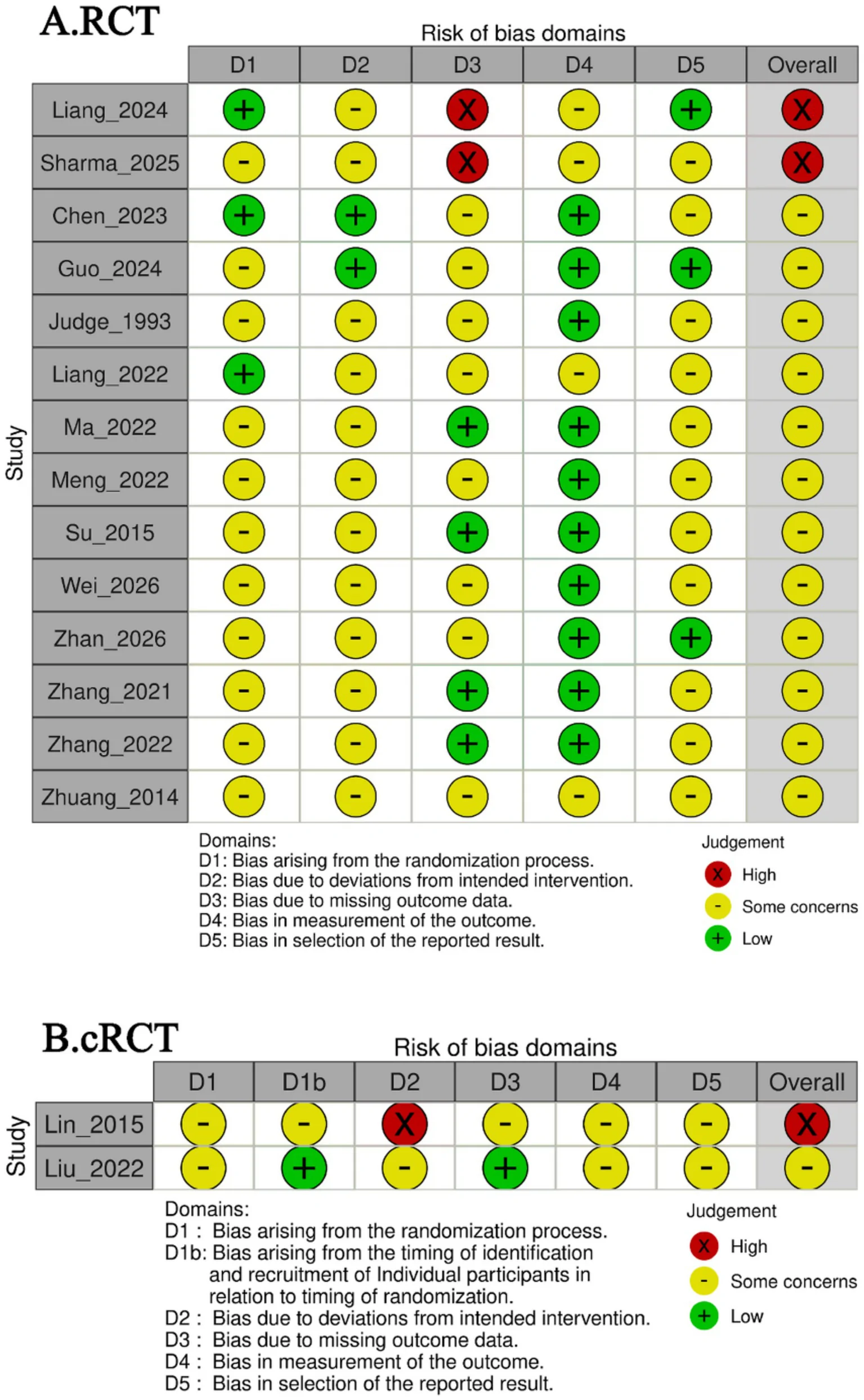
Risk-of-bias assessment for studies contributing to the quantitative synthesis. **(A)** Shows individually randomized controlled trials, and **(B)** shows cluster-randomized trials. D1 indicates bias arising from the randomization process; D1b indicates bias arising from the timing of identification or recruitment of individual participants in cluster-randomized trials; D2 indicates bias due to deviations from intended interventions; D3 indicates bias due to missing outcome data; D4 indicates bias in measurement of the outcome; D5 indicates bias in selection of the reported result. Green indicates low risk of bias, yellow indicates some concerns, and red indicates high risk of bias.

Risk of bias was assessed using the appropriate RoB 2 version. Figure 2 presents the study-level risk-of-bias judgments for the 16 studies contributing to quantitative synthesis, including 14 individually randomized controlled trials and two cluster-randomized controlled trials. Among the individually randomized trials, two were rated as having an overall High risk of bias and 12 as Some concerns; none was rated as overall Low. Among the cluster-randomized trials, one was rated as High and one as Some concerns. The main concerns related to the randomization process, deviations from intended interventions, missing outcome data, and selection of the reported result. Supplementary Figure S1 presents the summary distribution of RoB 2 judgments for the individually randomized trials.

### 3.4 Primary analysis of risk-domain composite SMDs and certainty of evidence

The primary analysis results for core-layer post-intervention risk-domain composite SMDs are shown in Table 2. Compared with non-exercise control, Tai Chi-based combined interventions favored the sarcopenia-related muscle function domain (SMD = 1.00, 95% CI: 0.12–1.88; k = 3; I^2^ = 24.5%) and the frailty/overall physical performance domain (SMD = 1.35, 95% CI: 0.38–2.31; k = 4; I^2^ = 77.1%). The muscle mass/quality domain remained uncertain (SMD = 0.60, 95% CI: −0.23–1.42; k = 2; I^2^ = 0%). Because all three bodies of evidence were rated Very low, these results supported favorable directions rather than definitive effects.

**Table 2:** Primary analysis of risk-domain composite SMDs.

Comparison type	Risk domain	k	SMD (95% CI)	I^2^	GRADE certainty	Main reasons for downgrading
Non-exercise control	Sarcopenia-related muscle function domain	3	0.999 (0.116 to 1.882)	24.50%	Very low	RoB, indirectness
	Sarcopenia-related muscle mass/quality domain	2	0.599 (−0.226 to 1.423)	0.00%	Very low	RoB, imprecision
	Frailty/overall physical performance domain	4	1.346 (0.382 to 2.309)	77.10%	Very low	RoB, inconsistency, indirectness
Active exercise control	Sarcopenia-related muscle function domain	4	0.217 (−0.423 to 0.857)	63.80%	Very low	RoB, inconsistency, indirectness, imprecision
	Sarcopenia-related muscle mass/quality domain	3	0.331 (−0.090 to 0.752)	0.00%	Low	RoB, imprecision
	Frailty/overall physical performance domain	3	0.626 (0.047 to 1.205)	0.00%	Very low	RoB, indirectness

This table presents the primary analysis of core-layer post-intervention risk-domain composite SMDs with k ≥ 2. SMD > 0 indicates results favoring Tai Chi-based combined interventions. The analysis assumed rho = 0.5 and used a REML random-effects model with Hartung–Knapp adjustment. Non-exercise control included usual care, health education, wait-list control, maintenance of usual activities, or blank control; active exercise control included other structured exercise interventions. Because the estimates combine related but non-identical measures, their magnitudes should be interpreted as standardized risk-domain effects rather than changes on a single clinical scale, together with the 95% CI, k, heterogeneity, key specific outcomes, and GRADE certainty. Fall-risk/balance-mobility domains with k < 2 were described narratively. Full forest plots are provided in Supplementary Figures S2–S5, GRADE judgments in Supplementary Table S4, and rho sensitivity analyses in Supplementary Table S5.

Compared with active exercise control, no stable additional benefit was observed in the sarcopenia-related muscle function domain (SMD = 0.22, 95% CI: −0.42–0.86; k = 4; I^2^ = 63.8%; Very low certainty) or the muscle mass/quality domain (SMD = 0.33, 95% CI: −0.09–0.75; k = 3; I^2^ = 0%; Low certainty). The frailty/overall physical performance domain showed a favorable point estimate (SMD = 0.63, 95% CI: 0.05–1.21; k = 3; I^2^ = 0%), but the certainty remained Very low and the lower confidence limit was close to the null, indicating uncertain additional benefit.

In the core layer, only 1 study provided post data for the fall-risk/balance-mobility domain under non-exercise control and active exercise control, respectively, which did not meet the threshold for quantitative synthesis. Therefore, this domain was not included in the main analysis table in the main text and was only interpreted narratively. Under active exercise control, although 2 studies in the core+extended layer were available for synthesis in this domain, the effect estimate was extremely imprecise and was therefore presented only as a supplementary result.

Overall, among the 6 synthesizable risk domains in the primary analysis, 1 was rated as Low and 5 as Very low, with no Moderate or High certainty evidence. The main reasons for downgrading included risk of bias, imprecision, indirectness of evidence, and inconsistency of results in some outcomes. The reporting/publication bias domain was not automatically downgraded because the number of studies in each individual meta-analysis pool did not reach k ≥ 10, and no formal funnel plot asymmetry test or Egger’s test was performed; this judgment does not indicate that publication bias was excluded. The full forest plots are provided in Supplementary Figures S2–S5, and the full GRADE judgments are provided in Supplementary Table S4.

### 3.5 Direct comparison with single Tai Chi and key specific outcomes

The results of direct comparisons with single Tai Chi and key specific outcomes are shown in Table 3. In the original data, only a small number of studies directly compared Tai Chi-based combined interventions with single Tai Chi or Tai Chi-derived controls, and the data forms were inconsistent. Therefore, no meta-analysis pool with k ≥ 2 using the same effect size model was formed. The current evidence therefore cannot quantitatively confirm that combined interventions have stable additional benefits compared with single Tai Chi.

**Table 3:** Specific outcome-pool sensitivity analysis results in the core layer.

Comparison type	Outcome/indicator	k	Effect size	I^2^
Non-exercise control	Grip strength	2	SMD = 1.199 (95% CI: −2.643 to 5.041)	32.38%
	Sit-to-stand ability	2	SMD = 1.000 (95% CI: 0.338 to 1.662)	0.00%
	SPPB	2	SMD = 1.458 (95% CI: −0.728 to 3.644)	0.00%
	Gait speed/gait ability	3	SMD = 1.272 (95% CI: −0.763 to 3.306)	83.94%
	Muscle mass/quality	2	SMD = 0.437 (95% CI: −0.276 to 1.151)	0.00%
Active exercise control	TUG	2	SMD = 0.232 (95% CI: −1.487 to 1.950)	0.00%
	Grip strength	4	SMD = 0.199 (95% CI: −0.429 to 0.828)	59.05%
	Muscle mass/quality	3	SMD = 0.143 (95% CI: −0.188 to 0.474)	0.00%

This table presents only the specific outcome-pool sensitivity analysis results in the core layer with k ≥ 2 and completed quantitative synthesis. SMD > 0 indicates results favoring Tai Chi-based combined interventions. Specific outcome-pool analyses were used to interpret the clinical sources of the primary analysis of risk-domain composite SMDs and do not replace the primary analysis of risk-domain composite SMDs. The direct comparison of TC-combined vs single-TC did not form a synthesizable meta-analysis pool with k ≥ 2 because of insufficient numbers of studies and inconsistencies in outcome indicators and data forms. Therefore, it was not included in this table and is described only narratively in the main text. The full specific outcome-pool results are provided in Supplementary Table S6.

Specific outcome-pool analyses supplemented the clinical interpretation of the risk-domain composite SMDs and did not replace the primary analysis. Compared with non-exercise control, sit-to-stand ability showed a favorable estimate (SMD = 1.00, 95% CI: 0.34–1.66; k = 2), whereas grip strength, SPPB, gait speed/gait ability, and muscle mass/quality remained affected by few studies, wide 95% CIs, or heterogeneity. Compared with active exercise control, grip strength, TUG, and muscle mass/quality did not show stable additional advantages. Some core+extended indicators showed favorable directions, but their interpretive priority was lower than that of the core-layer primary analysis. Full results are provided in Supplementary Table S6.

### 3.6 Robustness, supplementary analyses, and eligibility for publication bias assessment

The rho sensitivity analyses showed broadly consistent directions and interpretive boundaries under rho = 0.3, 0.5, and 0.7, and the core+extended analyses did not change the main conclusions. In the additional age-boundary analysis, excluding Sharma et al. changed the active-exercise-control frailty/overall physical performance estimate from SMD = 0.63 (95% CI: 0.05–1.21; k = 3) to SMD = 0.74 (95% CI: −2.64–4.11; k = 2), with I^2^ increasing from approximately 0 to 49.1%. The direction was unchanged, but precision deteriorated markedly. Only Meng et al. remained in the TUG pool, so no pooled estimate was calculated.

The GIV supplementary analysis and event data did not provide evidence sufficient to change the interpretation of the primary analysis. All meta-analysis pools available for eligibility assessment of publication bias testing had k < 10; therefore, Egger’s test, funnel plot asymmetry testing, and trim-and-fill were not performed. Thus, the absence of publication bias cannot be judged on this basis.

This age-boundary analysis evaluated the influence of retaining one study and did not isolate age as an independent effect modifier because participant-level age distributions and age-stratified outcomes were unavailable. The supplementary GRADE robustness check further showed that exclusion of studies with overall high risk of bias reduced synthesizability in some comparisons, while imprecision judgments for some muscle mass/quality and single muscle function outcomes were sensitive to threshold rules. These checks did not replace the primary analysis or main GRADE ratings. Full rho, specific-outcome, publication-bias eligibility, and GRADE robustness results are provided in Supplementary Tables S5–S8, and the age-boundary sensitivity analysis is shown in Supplementary Figure S6. The GIV and event-data analyses also did not alter the main interpretation.

### 3.7 Exploratory component description

The exploratory component description showed differences in effect direction and magnitude across combined forms and risk domains. However, most component–outcome–control combinations included only a few studies, and individual quantitative pools contained only 2–4 studies, with substantial differences in components, dose, participant status, and comparator conditions. Therefore, formal subgroup analysis or meta-regression was not considered sufficiently reliable. This analysis was not a network or component network meta-analysis and cannot identify an optimal combination, frequency, session duration, intervention period, or intervention ranking.

From the descriptive patterns, Tai Chi combined with resistance/strength training, endurance training, balance training, or multicomponent exercise showed potentially favorable directions in some risk domains. However, these findings can only provide hypotheses for subsequent head-to-head RCTs. Because the number of studies for each component combination was limited, and because participant criteria, intervention dose, and outcome measurements differed substantially, this study did not rank the effects of different combined structures.

## 4 Discussion

### 4.1 Main findings

Compared with non-exercise control, Tai Chi-based combined interventions showed favorable standardized effects in the sarcopenia-related muscle function domain (SMD = 1.00) and the frailty/overall physical performance domain (SMD = 1.35), whereas muscle mass/quality remained uncertain. These estimates were based on only 2–4 studies and Very low-certainty evidence. In addition, the composite SMDs integrated related but non-identical measures and therefore should not be interpreted as directly transferable changes on a single clinical scale. The evidence mainly supports potentially favorable directions rather than definitive clinical effects.

Compared with active exercise control, additional benefits were more uncertain. Muscle function and muscle mass/quality did not show stable advantages, whereas the frailty/overall physical performance domain showed a favorable point estimate (SMD = 0.63) based on three studies and Very low-certainty evidence. Excluding the age-boundary Sharma trial did not attenuate or reverse the point estimate (SMD = 0.74), but the confidence interval widened markedly when only two studies remained. This indicates reduced precision rather than evidence that age had no influence. Direct evidence against single Tai Chi also remained insufficient.

Overall, the findings should be interpreted within the certainty-of-evidence framework. Among 14 GRADE bodies of evidence, 2 were rated Low and 12 Very low, with none rated Moderate or High. The main limitations arose from risk of bias, imprecision, population and intervention indirectness, and inconsistency in some outcomes. Accordingly, the results support limited or uncertain trend-based judgments and do not establish clinical superiority, an optimal combined structure or dose, or a basis for strong recommendations.

### 4.2 Comparison with existing evidence on single Tai Chi and multicomponent exercise

Existing systematic reviews have mainly evaluated the effects of single Tai Chi on falls, balance, mobility, and physical performance in older adults (8, 9). The relevant evidence suggests that Tai Chi may improve balance- or mobility-related outcomes, such as TUG, functional reach, the Berg Balance Scale, single-leg stance, and gait speed, and may have some value for fall prevention in older adults (8). These studies provide a background for the application of Tai Chi in functional management among older adults. However, their core question remains whether “single Tai Chi is effective” (8, 9), and they cannot directly answer whether Tai Chi combined with resistance, strength, endurance, balance, or functional training provides additional benefits.

Recent evidence on digital Tai Chi has further focused on the effects of Tai Chi interventions delivered through videos, interactive platforms, or remote formats (48). This type of research suggests that changes in the delivery format of Tai Chi itself may not consistently improve muscle function or dynamic mobility (48). However, the study population still involved digital Tai Chi rather than combined interventions containing a Tai Chi component. Therefore, evidence on digital delivery cannot directly replace the evidence on “Tai Chi + other exercise or physical activity promotion components” examined in this study.

Evidence on multicomponent exercise suggests that the management of functional decline in older adults usually requires combined stimulation from strength, balance, endurance, and functional training (6, 10, 49). For older adults with sarcopenia, frailty, fall risk, or risk of reduced physical function, comprehensive training beyond a single exercise modality has public health plausibility (6, 10). The incremental contribution of this study is not to repeatedly examine whether single Tai Chi improves balance, nor is it equivalent to a general review of multicomponent exercise. Instead, this study evaluated intervention models in which the “Tai Chi component” was combined with resistance/strength, endurance, balance, or multicomponent training as an independent question.

Based on the findings of this study, Tai Chi-based combined interventions may provide some functional benefits compared with non-exercise control. However, compared with active exercise control or single Tai Chi, their additional benefits remain constrained by the lack of direct comparative studies and the limited GRADE certainty, and strong conclusions cannot yet be drawn.

### 4.3 Intervention structure, heterogeneity, and possible mechanisms

The favorable directions may reflect the Tai Chi component, the accompanying training component, and their combined functional stimuli, but the eligible interventions did not represent a standardized program. Tai Chi was combined with resistance or strength training, endurance exercise, balance training, functional gait training, Otago exercise, multicomponent exercise, or physical activity promotion. Intervention periods ranged from 4 to 52 weeks, frequency ranged from 2 to 5 weekly sessions to brief daily formats, and reported session durations ranged from approximately 20 to 90 min. Intensity, progression, supervision, adherence, and the relative contribution of each component were inconsistently reported, limiting attribution of effects to a specific structure.

The accompanying component may determine the principal physiological and functional stimulus. Resistance or strength training may be more relevant to weakness, grip strength, sit-to-stand performance, and lower-limb function (50, 51); endurance, walking, or functional training may be more relevant to activity tolerance, gait speed, 6MWT, and TUG; and balance or task-oriented training may be more directly related to BBS, SPPB, postural control, and fall-risk outcomes (49, 50). Tai Chi may additionally provide weight shifting, coordination, and postural-control stimuli (52, 53, 57). Nevertheless, the available studies did not permit reliable comparison of these combinations or determination of whether one component produced the observed effects reliably.

Comparator intensity also affects interpretation. Against non-exercise control, the observed effect may reflect Tai Chi, the accompanying exercise component, greater contact time, greater total training exposure, or their interaction. Active exercise controls provide a more direct test of additional benefit, but their effects were smaller and more uncertain. Sarcopenia and frailty involve multidimensional deficits in strength, muscle mass/quality, neuromuscular control, balance, gait, and activity tolerance (2, 3), providing a rationale for multicomponent approaches (10, 49). Nevertheless, because component categories were sparsely populated and GRADE certainty was Low or Very low, these mechanisms and component-specific explanations remain hypotheses rather than verified causal pathways, and the present evidence cannot identify an optimal combination or dose. This uncertainty also limits direct clinical translation across settings and populations within the currently available body of evidence.

### 4.4 Clinical and public health implications

The clinical and public health relevance of Tai Chi-based combined interventions lies in combining the accessibility and community feasibility of Tai Chi (7) with targeted resistance, strength, balance, endurance, or functional training (6, 10). The World Health Organization recommends multicomponent physical activity emphasizing functional balance and strength for older adults (10), while healthy-ageing initiatives emphasize maintaining functional ability (54). Within these frameworks, Tai Chi-based combined interventions may be regarded as a flexible candidate approach rather than a standardized program. Their use should be guided by functional screening, participant goals, safety, feasibility, and access to appropriate supervision.

From an implementation perspective, the accompanying component may be selected according to the predominant functional deficit. Resistance or strength training may be considered when muscle weakness is prominent; balance, gait, or task-oriented training may be relevant when falls or mobility limitations predominate; and endurance or walking components may be relevant when activity tolerance is limited. The observed programs ranged from 4 to 52 weeks, 2–5 weekly sessions or brief daily formats, and approximately 20–90 min per session, but these ranges should not be interpreted as an optimal dose. Frail participants or those with recurrent falls may require supervised initiation, progressive loading, balance support, and monitoring of adherence and adverse events.

It should be emphasized that the findings do not support directly elevating Tai Chi-based combined interventions to a strong recommendation or replacing established exercise programs. Favorable directions were observed for some muscle function and overall physical performance outcomes compared with non-exercise control, whereas additional benefits over active exercise or single Tai Chi remained uncertain and muscle mass/quality did not improve consistently. Given the Low or Very low certainty, the current clinical positioning should be an individualized candidate framework requiring further evaluation, rather than an established priority intervention or a fixed exercise prescription.

### 4.5 Methodological considerations, limitations, and future research directions

In the analytical design, this study attempted to distinguish different control types and used an approach combining risk-domain composite SMDs with key specific outcomes, in order to reduce the influence of repeated measurement of multiple related outcomes within the same study on pooled estimates. At the same time, the study process applied cluster correction for cRCTs and evaluated risk of bias and certainty of evidence using the RoB 2 tool, its cluster-randomized trial version, and the GRADE approach, thereby improving the transparency of result interpretation. However, these methodological procedures cannot eliminate the uncertainty arising from the insufficient number of original studies, limited study quality, and outcome heterogeneity.

This study has several limitations. First, most quantitative pools included only 2–4 studies, and direct comparisons with single Tai Chi were particularly sparse. Consequently, formal subgroup analysis or meta-regression by component, frequency, session duration, or intervention period would have been unstable, and no optimal combination or dose could be identified. Second, some outcomes had wide 95% CIs, and no pool reached k ≥ 10; therefore, funnel-plot asymmetry, Egger’s test, and trim-and-fill were not performed. Third, one trial enrolled women aged 55–70 years, although all study-arm mean ages exceeded 60 years. The original report described the recruited population as having a “history of risk of fall,” but the number of participants aged 55–59 years, the number with actual previous falls, individual fall-risk classifications, and age-stratified outcomes were unavailable. Group-level baseline measures of balance, mobility, and concern about falling supported its classification as risk-enriched age-boundary evidence, but did not establish that every participant had a high risk of falling. Excluding this trial did not reverse the point estimate but markedly reduced precision; therefore, age-specific effects could not be determined and some population indirectness remains. Fourth, participant status, diagnostic criteria, intervention components, training dose, comparators, and outcome measures varied, resulting in heterogeneity or indirectness. Fifth, overall risk of bias was High or Some concerns, and cluster correction for some cRCTs relied on ICC assumptions. Finally, composite SMDs reduced repeated weighting of related outcomes but combined different measures and constructs; they should therefore be interpreted as risk-domain effects rather than direct changes in TUG, SPPB, gait speed, grip strength, or another individual clinical measure.

Future studies should prioritize adequately powered and completely reported head-to-head RCTs comparing Tai Chi-based combined interventions with single Tai Chi and other active exercise programs. Trials should apply consistent sarcopenia, frailty, prefrailty, or functional-risk criteria and report age distributions, numbers below prespecified age thresholds, and age-stratified or interaction analyses when relevant. Intervention reports should specify the Tai Chi form, accompanying component, intensity, progression, frequency, session duration, total period, supervision, adherence, adverse events, and actual training exposure. More consistent clinical outcomes for muscle strength, muscle mass/quality, physical performance, balance, falls, and daily function are also needed. For cRCTs, the number of clusters, mean cluster size, ICC, design effect, and correction method should be reported (55). These improvements are required before the optimal combination, dose, clinical applicability, and additional value of Tai Chi-based combined interventions can be determined reliably.

## 5 Conclusion

This systematic review and meta-analysis suggests that Tai Chi-based combined interventions may improve selected muscle function and overall physical performance outcomes, particularly compared with non-exercise control. However, additional benefits over active exercise or single Tai Chi were uncertain, muscle mass/quality findings were inconsistent, and all GRADE bodies were rated Low or Very low. Accordingly, the evidence does not support a strong recommendation or a specific intervention prescription. Larger head-to-head RCTs with standardized population criteria, complete intervention reporting, clinically interpretable outcomes, and longer follow-up are needed.

## Data Availability

The datasets supporting the conclusions of this meta-analysis are included in the article and its Supplementary files. Search strategies and data extraction forms are available from the corresponding author upon reasonable request.
