# Supplementary material for: Effects of Tai Chi-based combined interventions on muscle function, muscle mass, and physical performance in older adults with sarcopenia, frailty, or risk of functional decline: a systematic review and meta-analysis
Source: Front Public Health. 2026 Aug 20;14:1891703. doi: 10.3389/fpubh.2026.1891703 (PMC13538022; doi:10.3389/fpubh.2026.1891703)
Supplement: Supplementary file 1 [file Supplementary_file_1.DOCX]

**Supplementary Table S1. PRISMA 2020 checklist**

| **Item no.** | **Section/topic** | **PRISMA 2020 checklist item** | **Reported location** | **Notes** |
| --- | --- | --- | --- | --- |
| 1 | Title | Identify the report as a systematic review. | Title page | The title identifies the study as a systematic review and meta-analysis. |
| 2 | Abstract | Report the abstract according to PRISMA 2020 for Abstracts. | Abstract | The structured abstract reports background, methods, results, conclusion, and registration. |
| 3 | Rationale | Explain the rationale for the review in the context of existing evidence. | Introduction 1.1–1.3 | The rationale for focusing on Tai Chi-based combined interventions is reported. |
| 4 | Objectives | State the review objective or research question clearly. | Introduction 1.4 | The review objective and comparison framework are reported. |
| 5 | Eligibility criteria | Specify inclusion and exclusion criteria and how studies were grouped for synthesis. | Methods 2.3; Results 3.2; Supplementary Table S3 | Age eligibility, core/extended population layers, comparator categories, and synthesis grouping are defined in Methods; characteristics of the age-boundary trial are reported in Results and Supplementary Table S3. |
| 6 | Information sources | Specify all databases, registers, and other sources searched, including the last search date. | Methods 2.2; Supplementary Table S2 | Databases, the trial registry, Chinese databases, and the last search date are reported. |
| 7 | Search strategy | Present the full search strategy for each database, register, or source. | Supplementary Table S2 | Full search strategies are provided for all searched sources. |
| 8 | Selection process | Describe how studies were screened and selected, including reviewer independence and conflict resolution. | Methods 2.2; Figure 1 | The screening process, independent assessment, and conflict-resolution procedures are reported. |
| 9 | Data collection process | Describe how data were extracted, including reviewer independence and cross-checking. | Methods 2.4 | Data-extraction and cross-checking procedures are reported. |
| 10a | Data items: outcomes | List and define all outcomes for which data were sought. | Methods 2.4; Supplementary Table S3 | Outcome domains and specific outcome pools are reported. |
| 10b | Data items: other variables | List and define other variables sought from the included studies. | Methods 2.4; Supplementary Table S3 | Study, participant, intervention, comparator, follow-up, and implementation variables are reported. |
| 11 | Study risk-of-bias assessment | Specify the methods and tools used to assess risk of bias in the included studies. | Methods 2.6; Figure 2; Supplementary Figure S1 | The RoB 2 methods are described in Methods 2.6. Study-level judgments for studies contributing to quantitative synthesis are presented in Figure 2, and the summary distribution for individually randomized trials is presented in Supplementary Figure S1. |
| 12 | Effect measures | Specify the effect measures used for each outcome. | Methods 2.5; Table 2 note; Supplementary Table S4 | SMD/Hedges’ g and event-effect measures are reported. |
| 13a | Synthesis methods: eligibility for synthesis | Describe how studies were judged eligible for each synthesis. | Methods 2.3–2.5 | Comparator type, population layer, risk-domain grouping, and the k ≥ 2 synthesis threshold are reported. |
| 13b | Synthesis methods: data preparation | Describe how data were prepared for synthesis. | Methods 2.4–2.5 | Direction harmonization, outcome mapping, composite SMD construction, and cRCT adjustment are reported. |
| 13c | Synthesis methods: display methods | Describe how study results and synthesis results were tabulated or visually displayed. | Methods 2.5; Tables 2–3; Supplementary Figures S2–S6 | Main results, supplementary and sensitivity analyses, and forest plots are reported. |
| 13d | Synthesis methods: statistical model | Describe the synthesis models and justify the model choices. | Methods 2.5 | The REML random-effects model, Hartung–Knapp adjustment, and heterogeneity statistics are reported. |
| 13e | Synthesis methods: heterogeneity | Describe the methods used to explore heterogeneity or possible effect modifiers. | Methods 2.5; Results 3.6–3.7; Supplementary Tables S5–S6; Supplementary Figure S6 | Correlation-coefficient, population-layer, specific-outcome, age-boundary, and descriptive component analyses are reported; formal subgroup analysis or meta-regression was not performed because quantitative pools contained only two to four studies. |
| 13f | Synthesis methods: sensitivity analyses | Describe the sensitivity analyses used to assess robustness. | Methods 2.5; Results 3.6; Supplementary Tables S5–S8; Supplementary Figure S6 | Correlation-coefficient, core versus core+extended, fixed-effect, leave-one-out, specific-outcome, GIV, event-data, age-boundary, and GRADE robustness analyses are reported. |
| 14 | Reporting bias assessment | Describe the methods used to assess reporting bias or small-study effects. | Methods 2.5; Results 3.6; Supplementary Table S7 | The k ≥ 10 criterion for funnel plots, Egger’s test, and the trim-and-fill method is reported. |
| 15 | Certainty assessment | Describe the methods used to assess certainty or confidence in the body of evidence. | Methods 2.6; Supplementary Table S4 | GRADE assessment methods and certainty judgments are reported. |
| 16a | Study selection: results | Report the results of the search and selection process. | Results 3.1; Figure 1 | The PRISMA flow is reported. |
| 16b | Study selection: excluded studies | Report potentially eligible studies excluded at the full-text stage and the reasons for exclusion. | Results 3.1; Figure 1 | Full-text exclusion reasons are summarized in the PRISMA flow diagram and Results section. |
| 17 | Study characteristics | Cite the included studies and present their characteristics. | Results 3.2; Table 1; Supplementary Table S3 | Study, participant, age, sex, condition, intervention, comparator, dose, and outcome characteristics are reported. |
| 18 | Risk of bias in studies | Present risk-of-bias assessments for the included studies. | Results 3.3; Figure 2; Supplementary Figure S1 | Study-level risk-of-bias judgments for studies contributing to quantitative synthesis are presented in Figure 2, and Supplementary Figure S1 presents the summary distribution of RoB 2 judgments for individually randomized trials. |
| 19 | Results of individual studies | Present summary statistics or effect estimates for individual studies where applicable. | Supplementary Figures S2–S6; Supplementary Table S6 | Study-level and pooled effects are reported for the primary, supplementary, and age-boundary sensitivity analyses where applicable. |
| 20a | Results of syntheses: contributing studies | Summarize the characteristics and risk of bias among studies contributing to each synthesis. | Results 3.4–3.6; Supplementary Table S4 | Certainty and major limitations are summarized by comparison and outcome. |
| 20b | Results of syntheses: statistical results | Present the results of all statistical syntheses conducted. | Tables 2–3; Supplementary Tables S5–S6; Supplementary Figures S2–S6 | Main, supplementary, specific-outcome, and age-boundary synthesis results are reported. |
| 20c | Results of syntheses: heterogeneity | Present the results of heterogeneity exploration or related analyses. | Results 3.6–3.7; Supplementary Tables S5–S6; Supplementary Figure S6 | Heterogeneity, outcome-specific sensitivity, age-boundary, and descriptive component analyses are reported. |
| 20d | Results of syntheses: sensitivity analyses | Present the results of sensitivity analyses. | Results 3.6; Supplementary Tables S5–S8; Supplementary Figure S6 | Correlation-coefficient, age-boundary, specific-outcome, GIV, event-data, and GRADE robustness analyses are reported. |
| 21 | Reporting biases | Present assessments of reporting bias or small-study effects for each synthesis assessed. | Results 3.6; Supplementary Table S7 | Formal tests were not performed because all meta-analysis pools had k < 10. |
| 22 | Certainty of evidence | Present the certainty assessment for each outcome or body of evidence assessed. | Results 3.4–3.6; Supplementary Table S4 | GRADE certainty is reported. |
| 23a | Discussion: interpretation | Provide a general interpretation of the results in the context of existing evidence. | Discussion 4.1–4.3 | Main findings are interpreted in relation to previous evidence and intervention mechanisms. |
| 23b | Discussion: limitations of evidence | Discuss limitations of the included evidence. | Discussion 4.5 | Limitations related to risk of bias, imprecision, indirectness, heterogeneity, and the inability to assess small-study effects are discussed. |
| 23c | Discussion: limitations of review process | Discuss limitations of the review methods and process. | Discussion 4.5 | Review-level limitations, including cRCT adjustment, sparse synthesis pools, and interpretation of composite SMDs, are discussed. |
| 23d | Discussion: implications | Discuss implications for practice, policy, or future research. | Discussion 4.4–4.5; Conclusion | Clinical implementation considerations, uncertainty regarding intervention combination and dose, safety considerations, and future research priorities are reported according to the certainty of evidence. |
| 24a | Registration and protocol | Provide registration information for the review. | Methods 2.1 | The PROSPERO registration number is reported. |
| 24b | Registration and protocol access | Indicate where the protocol or registration record can be accessed. | Methods 2.1 | The PROSPERO record is reported. |
| 24c | Protocol amendments | Describe and explain amendments or clarifications to the registered protocol. | Methods 2.1 and 2.5 | Before formal quantitative synthesis, the primary analytical level was clarified as risk-domain composite SMDs, with specific-outcome pools retained as sensitivity or supplementary analyses to reduce repeated weighting of multiple correlated outcomes within studies. This clarification did not alter the eligibility criteria or included studies. |
| 25 | Support | Describe financial or non-financial support. | Funding statement | The funding source and funder role are reported in the Funding statement. |
| 26 | Competing interests | Declare any competing interests. | Conflict of interest statement | Competing interests are reported in the Conflict of interest statement. |
| 27 | Availability of data, code, and materials | Report the availability of data, analytic code, and other materials. | Data availability statement; Supplementary Material | Data availability is reported in the Data availability statement. Extracted data, risk-of-bias judgments, GRADE evidence profiles, and analytic outputs are described in the article and Supplementary Material. |

**Supplementary Table S2. Complete search strategy**

The search date was May 12, 2026. The searched databases included PubMed, Embase, CENTRAL, Web of Science, Scopus, CINAHL, ClinicalTrials.gov, CNKI, Wanfang, and VIP. The search strategy consisted of terms related to Tai Chi, combined or multicomponent exercise, functional decline or sarcopenia/frailty in older adults, and randomized controlled trials. For English-language databases, controlled vocabulary terms and free-text terms were combined. For Chinese-language databases, searches were conducted using combinations of subject, title, keyword, or abstract fields. The complete search strategies for each database are shown below. The number of records retrieved and the screening process are presented in Figure 1.

| **Database** | **Search strategy** |
| --- | --- |
| PubMed | (“Tai Ji”[Mesh] OR “tai chi”[tiab] OR taichi[tiab] OR “tai ji”[tiab] OR taiji[tiab] OR taijiquan[tiab] OR “tai ji quan”[tiab] OR “tai chi chuan”[tiab] OR “taiji quan”[tiab]) AND (“Resistance Training”[Mesh] OR “Endurance Training”[Mesh] OR “multicomponent exercise”[tiab] OR “multi-component exercise”[tiab] OR “combined exercise”[tiab] OR “combined training”[tiab] OR “resistance training”[tiab] OR “strength training”[tiab] OR “balance training”[tiab] OR “functional training”[tiab] OR “gait training”[tiab] OR “aerobic training”[tiab] OR “exercise snack”[tiab] OR “exercise snacking”[tiab]) AND (“Aged”[Mesh] OR “Frail Elderly”[Mesh] OR “Sarcopenia”[Mesh] OR “Frailty”[Mesh] OR “Accidental Falls”[Mesh] OR “older adult”[tiab] OR elderly[tiab] OR sarcopeni*[tiab] OR frail*[tiab] OR “fall risk”[tiab] OR “functional decline”[tiab] OR “low muscle strength”[tiab] OR “slow gait”[tiab]) AND (“Randomized Controlled Trial”[pt] OR randomized[tiab] OR randomised[tiab] OR randomly[tiab] OR “random allocation”[tiab] OR trial[ti] OR “clinical trial”[tiab] OR “cluster randomized”[tiab]) NOT (animals[mh] NOT humans[mh]) |
| Embase | (‘Tai Chi’/exp OR ‘tai chi’:ti,ab,kw OR taichi:ti,ab,kw OR ‘tai ji’:ti,ab,kw OR taiji:ti,ab,kw OR taijiquan:ti,ab,kw) AND (‘resistance training’/exp OR ‘endurance training’/exp OR ‘multicomponent exercise’:ti,ab,kw OR ‘combined exercise’:ti,ab,kw OR ‘combined training’:ti,ab,kw OR ‘resistance training’:ti,ab,kw OR ‘strength training’:ti,ab,kw OR ‘balance training’:ti,ab,kw OR ‘functional training’:ti,ab,kw OR ‘gait training’:ti,ab,kw OR ‘exercise snacking’:ti,ab,kw) AND (‘aged’/exp OR ‘frail elderly’/exp OR ‘sarcopenia’/exp OR ‘frailty’/exp OR ‘falling’/exp OR ‘older adult’:ti,ab,kw OR elderly:ti,ab,kw OR sarcopeni*:ti,ab,kw OR frail*:ti,ab,kw OR ‘fall risk’:ti,ab,kw OR ‘functional decline’:ti,ab,kw OR ‘low muscle strength’:ti,ab,kw) AND (‘randomized controlled trial’:it OR randomized:ti,ab,kw OR randomised:ti,ab,kw OR ‘random allocation’:ti,ab,kw OR trial:ti OR ‘clinical trial’:ti,ab,kw OR ‘cluster randomized’:ti,ab,kw) NOT (‘animal’/exp NOT ‘human’/exp) |
| CENTRAL | (“tai chi” OR taichi OR “tai ji” OR taiji OR taijiquan OR “tai ji quan” OR “tai chi chuan” OR “taiji quan”):ti,ab,kw AND (“multicomponent exercise” OR “multi-component exercise” OR “combined exercise” OR “combined training” OR “resistance training” OR “strength training” OR “balance training” OR “functional training” OR “gait training” OR “aerobic training” OR “exercise snack” OR “exercise snacking”):ti,ab,kw AND (“older adult” OR elderly OR aged OR sarcopeni* OR frail* OR prefrail OR “fall risk” OR “functional decline” OR “low physical function” OR “low muscle strength” OR “slow gait”):ti,ab,kw |
| Web of Science | TS=(“tai chi” OR taichi OR “tai ji” OR taiji OR taijiquan OR “tai ji quan” OR “tai chi chuan” OR “taiji quan”) AND TS=(“multicomponent exercise” OR “multi-component exercise” OR “combined exercise” OR “combined training” OR “resistance training” OR “strength training” OR “balance training” OR “functional training” OR “gait training” OR “aerobic training” OR “exercise snack” OR “exercise snacking”) AND TS=(“older adult” OR elderly OR aged OR sarcopeni* OR frail* OR prefrail OR “fall risk” OR “functional decline” OR “low physical function” OR “low muscle strength” OR “slow gait”) AND TS=(randomized OR randomised OR randomly OR “random allocation” OR trial OR “clinical trial” OR “cluster randomized”) |
| Scopus | TITLE-ABS-KEY(“tai chi” OR taichi OR “tai ji” OR taiji OR taijiquan OR “tai ji quan” OR “tai chi chuan” OR “taiji quan”) AND TITLE-ABS-KEY(“multicomponent exercise” OR “multi-component exercise” OR “combined exercise” OR “combined training” OR “resistance training” OR “strength training” OR “balance training” OR “functional training” OR “gait training” OR “aerobic training” OR “exercise snack” OR “exercise snacking”) AND TITLE-ABS-KEY(“older adult” OR elderly OR aged OR sarcopeni* OR frail* OR prefrail OR “fall risk” OR “functional decline” OR “low physical function” OR “low muscle strength” OR “slow gait”) AND TITLE-ABS-KEY(randomized OR randomised OR randomly OR “random allocation” OR trial OR “clinical trial” OR “cluster randomized”) |
| CINAHL | ((MH “Tai Chi”) OR TI/AB (“tai chi” OR taichi OR “tai ji” OR taiji OR taijiquan)) AND ((MH “Resistance Training”) OR (MH “Endurance Training”) OR TI/AB (“multicomponent exercise” OR “combined exercise” OR “resistance training” OR “strength training” OR “balance training” OR “functional training” OR “gait training” OR “exercise snacking”)) AND ((MH “Aged+”) OR (MH “Frail Elderly”) OR (MH “Sarcopenia”) OR TI/AB (“older adult” OR elderly OR sarcopeni* OR frail* OR “fall risk” OR “functional decline” OR “low muscle strength”)) AND (PT randomized controlled trial OR PT clinical trial OR TI/AB randomized OR randomised OR “cluster randomized”) |
| ClinicalTrials.gov | (“tai chi” OR taichi OR “tai ji” OR taiji OR taijiquan OR “tai ji quan” OR “tai chi chuan” OR “taiji quan”) AND (“multicomponent exercise” OR “multi-component exercise” OR “combined exercise” OR “combined training” OR “resistance training” OR “strength training” OR “balance training” OR “functional training” OR “exercise snack” OR “exercise snacking”) AND (“older adult” OR elderly OR aged OR sarcopenia OR frailty OR prefrail OR “fall risk” OR “functional decline” OR “low muscle strength”) AND (randomized OR randomised OR randomly OR “random allocation” OR trial OR “clinical trial” OR “cluster randomized”) |
| CNKI | #1：太极 + 太极拳 + 太极运动 + 太极训练 + 太极拳运动 + 简化太极拳 + 二十四式太极 + 24式太极；  #2：老年 + 老年人 + 高龄 + 肌少症 + 少肌症 + 肌肉减少症 + 衰弱 + 虚弱 + 跌倒 + 跌倒风险 + 平衡 + 肌力 + 步速 + 身体功能；  #3：随机 + 随机对照 + 随机分组 + 随机试验 + 随机对照试验 + 临床试验 + 对照试验 + RCT；  #4：#1 AND #2 AND #3 |
| Wanfang | #1：太极 OR 太极拳 OR 太极运动 OR 太极训练 OR 太极拳运动 OR 简化太极拳 OR 二十四式太极 OR 24式太极；  #2：老年 OR 老年人 OR 高龄 OR 肌少症 OR 少肌症 OR 肌肉减少症 OR 衰弱 OR 虚弱 OR 跌倒 OR 跌倒风险 OR 平衡 OR 肌力 OR 步速 OR 身体功能；  #3：随机 OR 随机对照 OR 随机分组 OR 随机试验 OR 随机对照试验 OR 临床试验 OR 对照试验 OR RCT；  #4：#1 AND #2 AND #3 |
| VIP | #1：M/R=(太极 OR 太极拳 OR 太极运动 OR 太极训练 OR 太极拳运动 OR 简化太极拳 OR 二十四式太极 OR 24式太极)；  #2：M/R=(老年 OR 老年人 OR 高龄 OR 肌少症 OR 少肌症 OR 肌肉减少症 OR 衰弱 OR 虚弱 OR 跌倒 OR 跌倒风险 OR 平衡 OR 肌力 OR 步速 OR 身体功能)；  #3：M/R=(随机 OR 随机对照 OR 随机分组 OR 随机试验 OR 随机对照试验 OR 临床试验 OR 对照试验 OR RCT)；  #4：#1 AND #2 AND #3 |

**Supplementary Table S3. Detailed eligibility, participant, intervention, and outcome characteristics of the included reports**

| **Study/report** | **Eligibility/diagnostic criteria** | **Group-level participant data** | **Intervention details** | **Comparator details** | **Training dose** | **Exact outcomes/instruments** |
| --- | --- | --- | --- | --- | --- | --- |
| Ma Zuoyao 2022 | Age ≥60 years; no long-term exercise or Tai Chi history; no severe disease affecting exercise; willing to complete testing | TC+ST: R 50/A NR, age 68.41; TC: 50/NR, age 68.15; ST: 47/NR, age 68.62; NEC: 45/NR, age 66.69; female NR | 24-form Tai Chi plus five core-strength exercises | Single Tai Chi; core-strength training; usual lifestyle | ≥4 sessions/week; Tai Chi ≥30 min; core strength approximately 40 min; combined components ≥30 min each; 24 weeks | Gait speed; TUG; 6MWT; Tetrax fall-risk index |
| Ma Zuoyao 2020 | Age ≥60 years; no long-term exercise or Tai Chi history; no severe disease affecting exercise; willing to complete testing | TC+ST: R 50/A NR, age 68.41; TC: 50/NR, age 68.15; ST: 47/NR, age 68.62; NEC: 45/NR, age 66.69; female NR | 24-form Tai Chi plus five core-strength exercises | Single Tai Chi; core-strength training; usual lifestyle | ≥4 sessions/week; combined group ≥60 min; Tai Chi ≥30 min; core strength approximately 40 min; 24 weeks | Lumbar spine BMD; BBS |
| Zhang Junpeng 2021 | Age 60–75 years; regular Tai Chi ≥150 min/week for ≥1 year; healthy; independent ADL; normal cognition | Combined: R 30/A 28, age 67.4±6.1, female 12/28; TC: 30/28, age 66.6±5.9, female 11/28 | Habitual Tai Chi plus aerobic and resistance training | Habitual Tai Chi alone | Additional training 2 sessions/week; Tai Chi ≥3 sessions/week and ≥30 min/session; 12 weeks | Grip strength; 30-s chair stand; 10-m maximum gait speed; TUG |
| Judge 1993 | Older women who passed medical examination and exercise testing; symptomatic lower-extremity arthritis allowed when passive ROM was unrestricted | Overall R 38/A 21; arm-specific n NR; age 67.8 and 68.5 in the reported groups; female 100% | Simple Tai Chi movements plus resistance, walking, flexibility, and postural exercises | Postural-control and flexibility exercise | Combined intervention 3 sessions/week; comparator 1 session/week after week 13; session duration not fully reported; 24 weeks | Single-leg stance centre-of-force displacement |
| Wolfson 1996 | Community-dwelling adults aged ≥75 years who passed medical and functional screening | EDU: R 27/A NR, age 80.6; BT: 28/NR, age 78.9; ST: 28/NR, age 80.0; BT+ST: 27/NR, age 79.7; female 42% overall | Balance and/or progressive lower-extremity strength training followed by Tai Chi maintenance | Education and usual activity | Three sessions/week for 13 weeks; subsequently Tai Chi once/week plus home practice for 26 weeks; intensive sessions 45 or 90 min; Tai Chi 60 min; 39 weeks | Sensory Organization Testing losses of balance; single-stance time; functional base of support; isokinetic torque; usual gait velocity |
| Zhang 2022 | Age 65±5 years; stable health; independent living and walking; reinforced Romberg score ≥41; MoCA ≥26 | Intervention: R 9/A NR; control: 9/NR; overall age 63; female 10/18 | Simplified 24-form Tai Chi with somatosensory interactive balance exergame | Matched physical exercise | ≥3 sessions/week; 20 min/session; 4 weeks | Gait speed; standing balance; 5TCS; TUG; letter-digit test; Stroop test |
| Chen 2023 | AWGS 2019 sarcopenia; age ≥60 years; able to complete TUG or 5TCS independently; no supervised exercise during the previous 3 months | Intervention: R 30/A 25, age 65.68; control: 30/24, age 65.21; female 100% | Simplified 24-form Tai Chi plus progressive elastic-band resistance training | Health education and no exercise | 3 sessions/week; 60 min/session; 8 weeks | ASM; SMM; SMI; handgrip strength; gait speed; 5TCS |
| Sharma 2025 | Women aged 55–70 years; independently able to stand, walk, and rise from a chair; able to follow instructions; the original report described a “history of risk of fall,” but the number with actual previous falls, individual fall-risk classifications, the number aged 55–59 years, and age-stratified outcomes were not reported; classified as risk-enriched age-boundary evidence | OEP: R 15/A 10, age 62.0±4.59; OEP+TC: 15/10, age 64.40±3.37; walking: 15/10, age 64.30±5.05; female 100% | Otago Exercise Programme plus Sun-style Tai Chi | Otago Exercise Programme; walking control | 3 sessions/week; 80 min/session, comprising 50 min OEP and 30 min Tai Chi; 8 weeks | TUG; BBS; FES-I |
| Meng 2022 | Age ≥65 years; Fried phenotype score 3–5; able to walk ≥6 min without assistance; no exercise training during the previous year | TC: R NR/A 50, age 75.60±1.92, female 25/50; combined: NR/50, age 76.31±2.07, female 30/50; RT+ET: NR/50, age 76.59±2.58, female 29/50 | Eight-form Yang-style Tai Chi plus strength and endurance exercise | Single Tai Chi; strength and endurance exercise | 3 sessions/week; approximately 90 min/session, including warm-up and cool-down; 24 weeks | 10-m maximum walking speed; grip strength; TUG; 6MWT; frailty reversal |
| Rikkonen 2023 | Home-dwelling women born in 1932–1945 and living near Kuopio; long-term institutional care excluded | Intervention: R 457/A NR, age 76.4; control: 457/NR, age 76.6; female 100% | Gym-based strength training plus classic Tai Chi | Fall-prevention education and usual activity | One gym-based session plus one Tai Chi session/week; 60 min each; 26-week supervised phase | Falls; injurious falls; fractures |
| Woo 2024 | Age ≥70 years; at least one frailty indicator; able to communicate; consent provided | Intervention: R 21/A 16, age 84.31±9.21; control: 22/18, age 84.38±9.01; all participants male | Tai Chi plus resistance-band training | Frailty-related health education | 3 sessions/week; 60 min/session; 12 weeks | Frailty index; IADL; back-scratch test; arm-curl test; physical activity; quality of life; HRV |
| Zhan 2026 | Age 60–75 years; SARC-CalF ≥11; low grip strength and ASM; SPPB ≤9; MMSE >17; able to undertake training | HEG: R 16/A 16, age 71.75±3.17, female 10/16; EBG: 17/17, age 71.59±3.50, female 10/17; CG: 16/16, age 70.31±2.30, female 9/16 | 24-form Tai Chi plus elastic-band resistance training | Elastic-band resistance training; WeChat health education without physical training | 3 sessions/week; EBG 40 min/session; HEG 70 min/session; 12 weeks | Grip strength; ASM; SPPB; Stroop reaction time and accuracy; prefrontal oxyhaemoglobin |
| Su 2015 | Explicit eligibility criteria NR | NEC: R 16/A NR; TC: 17/NR; weighted TC: 16/NR; overall age 65.3; female 7/16, 7/17, and 8/16 | Weighted-vest Tai Chi | Single Tai Chi; no-intervention control | 4–5 sessions/week; 60 min/session; 16 weeks | Knee-extensor, knee-flexor, ankle-plantarflexor, and ankle-dorsiflexor peak torque |
| Alegre-Tamariz 2025 | Age ≥60 years; non-institutionalized; MMSE ≥24; able to stand and walk ≥10 m without assistance; Barthel Index ≥60 | Intervention: R 29/A NR; control: 18/NR; overall age 72.66±7.27; female 45/47 | Physical-activity workshops incorporating Tai Chi plus functional gait training | Physical-activity workshops alone | Functional gait training 3 sessions/week plus weekly workshops; 30–40 min/session; 12 weeks | TUG; handgrip strength; calf circumference; Barthel Index |
| Liang 2022 | Age ≥65 years; no regular structured exercise; GFI ≤4; passed functional safety screening | ES: R 15/A NR; TCS: 16/NR; ES+TCS: 15/NR; control: 17/NR; age 71.1, 72.6, 73.3, and 71.9; female 54% overall | Exercise snacking plus Chen-style Tai Chi snacking | Exercise snacking; Tai Chi snacking; web-based physical-activity information | Daily; combined group approximately 20 min/day as two approximately 10-min sessions; 4 weeks | 60-s chair-rise repetitions; five-repetition sit-to-stand time; single-leg balance |
| Yang 2025 | Age 60–75 years; medically stable; independent ambulation for more than one city block; no previous Tai Chi or Roliball training | TC+RB: R 45/A 45, age 66.98±6.36, female 30/45; basic TC: 45/45, age 66.87±2.98, female 30/45; 24-form TC: 45/45, age 67.98±7.29, female 31/45 | Yang-style Tai Chi plus Roliball demonstration | Basic Tai Chi; 24-form Tai Chi | 3 sessions/week; 90 min/session; 12 weeks | TUG; gait speed; BBS; MoCA; one-leg standing test; chair-stand test; modified FES; handgrip strength; Stroop Color and Word Test |
| Guo 2024 | Age 60–75 years; sarcopenia diagnosed according to AWGS 2016 | TC+ST: R NR/A 33, age 66.94±4.42, female 19/33; ST: NR/30, age 66.87±3.84, female 17/30; education control: NR/30, age 65.42±3.97, female 14/30 | Tai Chi plus progressive resistance/strength training | Strength training; education control | 3 sessions/week; 90 min/session; 24 weeks | L3 skeletal muscle area; skeletal muscle density; skeletal muscle fat indices; RSMI; grip strength |
| Zhuang 2014 | Age 60–80 years; community dwelling; completed baseline assessment | Intervention: R 28/A 22, age 66.3, female 15/22; control: 28/28, age 65.4, female 21/28 | Eight-form Yang-style Tai Chi plus balance, body-weight strength, and flexibility training | Maintenance of usual physical activity | 3 sessions/week; 60 min/session; 12 weeks | TUG; 30-s chair stand; Star Excursion Balance Test; isokinetic strength; spatiotemporal gait; joint ROM |
| Liu 2022 | Age ≥75 years; Fried phenotype score 1–2; able to communicate and ambulate without personal assistance | Intervention: R 72/A 67, age 80.75; control: 71/68, age 80.74; female 70.37% overall | Tai Chi plus strength and mobility exercises | Routine care and usual daily activities | 5 sessions/week; 40 min planned and 37.6 min actual mean duration; 52 weeks | Fried frailty phenotype score; gait velocity and related gait parameters |
| Lin 2015 | Age ≥65 years; no cognitive impairment; able to follow instructions and walk without assistance | Intervention: R 54/A 48, age 73.9, female 30/48; control: 54/47, age 74.2, female 31/47 | Ten simplified Tai Chi forms plus concurrent Thera-Band resistance exercise | Routine sedentary day-care activities | 2 sessions/week; 60 min/session; 16 weeks | Senior Fitness Test; 6MWT; 8-foot up-and-go test; manual muscle strength |
| Wei 2026 | Age >60 and <75 years; sarcopenia diagnosed according to AWGS 2016 | Hybrid: R NR/A 31, age 66.19±5.00, female 15/31; RT: NR/30, age 66.87±3.84, female 17/30; education: NR/30, age 65.42±3.97, female 14/30 | Self-determined sequencing of Tai Chi, resistance, or hybrid training | Resistance training; education and telephone follow-up | 3 sessions/week; approximately 90 min including warm-up and cool-down; 24 weeks | L3 skeletal muscle area; skeletal muscle density; muscle fat infiltration; handgrip strength; RSMI; sarcopenia reversal |
| Liang 2024 | Age ≥65 years; SPPB strength and balance domain score 2–6/8; not regularly exercising; considered safe for training | Intervention: R 44/A NR; wait-list: 46/NR; overall age 74.1; female 31/44 and 33/46 | Progressive strength snacking plus Tai Chi snacking | Usual lifestyle and wait-list control | Two exercise snacks/day, one strength and one Tai Chi; approximately 10 min/snack; 12 weeks | SPPB; 60-s sit-to-stand; single-leg balance; TUG; peak force |

Note: Data are presented as mean±SD, mean, n/N (%), or range, as reported in the original studies. R/A indicates randomized/analyzed sample size. NR indicates not reported. Ma Zuoyao 2020 and Ma Zuoyao 2022 originated from the same randomized sample and were counted as one independent study.

Abbreviations: 5TCS, five-times sit-to-stand test; 6MWT, six-minute walk test; ADL, activities of daily living; ASM, appendicular skeletal muscle mass; AWGS, Asian Working Group for Sarcopenia; BBS, Berg Balance Scale; BMD, bone mineral density; CG, control group; EBG, elastic-band group; EDU, education control; ES, exercise snacking; FES, Falls Efficacy Scale; FES-I, Falls Efficacy Scale–International; GFI, Groningen Frailty Indicator; HEG, hybrid exercise group; HRV, heart rate variability; IADL, instrumental activities of daily living; MMSE, Mini-Mental State Examination; MoCA, Montreal Cognitive Assessment; NEC, non-exercise control; OEP, Otago Exercise Programme; ROM, range of motion; RSMI, relative skeletal muscle index; RT, resistance training; SARC-CalF, SARC-F combined with calf circumference; SMI, skeletal muscle index; SMM, skeletal muscle mass; SPPB, Short Physical Performance Battery; ST, strength training; TC, Tai Chi; TCS, Tai Chi snacking; TUG, Timed Up and Go test.

**Supplementary Table S4. GRADE evidence profile and certainty-of-evidence judgments**

| **Evidence level** | **Comparison type** | **Clinical evidence domain** | **Outcome** | **k** | **SMD (95%CI)** | **I^2^** | **GRADE certainty** | **Main reasons for downgrading** | **Interpretation** |
| --- | --- | --- | --- | --- | --- | --- | --- | --- | --- |
| Key specific outcome | Active exercise control | Sarcopenia-related muscle function domain | Grip strength | 4 | 0.199 (-0.429 to 0.828) | 59.05% | Very low | RoB downgraded by 1 level; inconsistency downgraded by 1 level; imprecision downgraded by 1 level | Suggested only a possible trend and did not support a definitive effect conclusion |
| composite SMD primary analysis | Active exercise control | Sarcopenia-related muscle function domain | Sarcopenia-related muscle function domain | 4 | 0.217 (-0.423 to 0.857) | 63.80% | Very low | RoB downgraded by 1 level; inconsistency downgraded by 1 level; indirectness downgraded by 1 level; imprecision downgraded by 1 level | Suggested only a possible trend and did not support a definitive effect conclusion |
| Key specific outcome | Active exercise control | Sarcopenia-related muscle mass/quality domain | Muscle mass/quality | 3 | 0.143 (-0.188 to 0.474) | 0.00% | Low | RoB downgraded by 1 level; imprecision downgraded by 1 level | May be interpreted as limited evidence and should be expressed cautiously |
| composite SMD primary analysis | Active exercise control | Sarcopenia-related muscle mass/quality domain | Sarcopenia-related muscle mass/quality domain | 3 | 0.331 (-0.090 to 0.752) | 0.00% | Low | RoB downgraded by 1 level; imprecision downgraded by 1 level | May be interpreted as limited evidence and should be expressed cautiously |
| Key specific outcome | Active exercise control | Frailty/overall physical performance domain | TUG | 2 | 0.232 (-1.487 to 1.950) | 0.00% | Very low | RoB downgraded by 2 levels; imprecision downgraded by 2 levels | Suggested only a possible trend and did not support a definitive effect conclusion |
| composite SMD primary analysis | Active exercise control | Frailty/overall physical performance domain | Frailty/overall physical performance domain | 3 | 0.626 (0.047 to 1.205) | 0.00% | Very low | RoB downgraded by 2 levels; indirectness downgraded by 1 level | Suggested only a possible trend and did not support a definitive effect conclusion |
| Key specific outcome | Non-exercise control | Sarcopenia-related muscle function domain | Grip strength | 2 | 1.199 (-2.643 to 5.041) | 32.38% | Very low | RoB downgraded by 1 level; imprecision downgraded by 2 levels | Suggested only a possible trend and did not support a definitive effect conclusion |
| Key specific outcome | Non-exercise control | Sarcopenia-related muscle function domain | Sit-to-stand ability | 2 | 1.000 (0.338 to 1.662) | 0.00% | Very low | RoB downgraded by 2 levels; imprecision downgraded by 1 level | Suggested only a possible trend and did not support a definitive effect conclusion |
| composite SMD primary analysis | Non-exercise control | Sarcopenia-related muscle function domain | Sarcopenia-related muscle function domain | 3 | 0.999 (0.116 to 1.882) | 24.50% | Very low | RoB downgraded by 2 levels; indirectness downgraded by 1 level | Suggested only a possible trend and did not support a definitive effect conclusion |
| Key specific outcome | Non-exercise control | Sarcopenia-related muscle mass/quality domain | Muscle mass/quality | 2 | 0.437 (-0.276 to 1.151) | 0.00% | Very low | RoB downgraded by 1 level; imprecision downgraded by 2 levels | Suggested only a possible trend and did not support a definitive effect conclusion |
| composite SMD primary analysis | Non-exercise control | Sarcopenia-related muscle mass/quality domain | Sarcopenia-related muscle mass/quality domain | 2 | 0.599 (-0.226 to 1.423) | 0.00% | Very low | RoB downgraded by 1 level; imprecision downgraded by 2 levels | Suggested only a possible trend and did not support a definitive effect conclusion |
| Key specific outcome | Non-exercise control | Frailty/overall physical performance domain | SPPB | 2 | 1.458 (-0.728 to 3.644) | 0.00% | Very low | RoB downgraded by 2 levels; imprecision downgraded by 2 levels | Suggested only a possible trend and did not support a definitive effect conclusion |
| Key specific outcome | Non-exercise control | Frailty/overall physical performance domain | Gait speed/gait ability | 3 | 1.272 (-0.763 to 3.306) | 83.94% | Very low | RoB downgraded by 2 levels; inconsistency downgraded by 2 levels; imprecision downgraded by 1 level | Suggested only a possible trend and did not support a definitive effect conclusion |
| composite SMD primary analysis | Non-exercise control | Frailty/overall physical performance domain | Frailty/overall physical performance domain | 4 | 1.346 (0.382 to 2.309) | 77.10% | Very low | RoB downgraded by 2 levels; inconsistency downgraded by 2 levels; indirectness downgraded by 1 level | Suggested only a possible trend and did not support a definitive effect conclusion |

Note: SMD > 0 indicates results favoring Tai Chi-based combined interventions. GRADE certainty of evidence was assessed at the outcome level, with downgrading domains including risk of bias, inconsistency, indirectness, imprecision, and publication bias. RoB indicates risk of bias; TUG, Timed Up and Go test; SPPB, Short Physical Performance Battery. The GRADE assessment generated 14 bodies of evidence, of which 2 were rated as Low and 12 as Very low, with no High or Moderate evidence identified. Low certainty indicates that the current evidence is limited and still requires cautious interpretation in combination with the 95%CI, number of studies, and risk of bias; Very low certainty indicates only a possible trend or insufficient current evidence and should not be used to form definitive conclusions or strong recommendations. The reporting bias/publication bias domain was not automatically downgraded because no individual meta-analysis pool reached k ≥ 10, and formal funnel plot asymmetry testing, Egger’s test, or trim-and-fill analysis was not performed; this judgment does not indicate that publication bias was excluded. The composite SMD primary analysis served as the main basis for interpretation in the main text, whereas key specific outcomes were used to illustrate the clinical sources of domain-level effects and did not replace the risk-domain composite SMD primary analysis.

**Supplementary Table S5. Summary of composite SMD sensitivity analyses**

| **Comparison type** | **Data model** | **Analysis level** | **Risk domain** | **rho** | **Analysis position** | **k** | **SMD (95%CI)** | **I^2^** |
| --- | --- | --- | --- | --- | --- | --- | --- | --- |
| Non-exercise control | post | core | Sarcopenia-related muscle function domain | 0.3 | Sensitivity analysis: low-correlation assumption | 3 | 0.995 (0.113 to 1.877) | 24.40% |
|  | post | core | Sarcopenia-related muscle function domain | 0.5 | Primary-analysis reference | 3 | 0.999 (0.116 to 1.882) | 24.50% |
|  | post | core | Sarcopenia-related muscle function domain | 0.7 | Sensitivity analysis: high-correlation assumption | 3 | 1.004 (0.121 to 1.886) | 24.70% |
|  | post | core | Sarcopenia-related muscle mass/quality domain | 0.3 | Sensitivity analysis: low-correlation assumption | 2 | 0.599 (-0.226 to 1.423) | 0.00% |
|  | post | core | Sarcopenia-related muscle mass/quality domain | 0.5 | Primary-analysis reference | 2 | 0.599 (-0.226 to 1.423) | 0.00% |
|  | post | core | Sarcopenia-related muscle mass/quality domain | 0.7 | Sensitivity analysis: high-correlation assumption | 2 | 0.599 (-0.226 to 1.423) | 0.00% |
|  | post | core | Frailty/overall physical performance domain | 0.3 | Sensitivity analysis: low-correlation assumption | 4 | 1.342 (0.381 to 2.303) | 77.00% |
|  | post | core | Frailty/overall physical performance domain | 0.5 | Primary-analysis reference | 4 | 1.346 (0.382 to 2.309) | 77.10% |
|  | post | core | Frailty/overall physical performance domain | 0.7 | Sensitivity analysis: high-correlation assumption | 4 | 1.350 (0.384 to 2.316) | 77.20% |
| Active exercise control | post | core | Sarcopenia-related muscle function domain | 0.3 | Sensitivity analysis: low-correlation assumption | 4 | 0.217 (-0.423 to 0.857) | 63.70% |
|  | post | core | Sarcopenia-related muscle function domain | 0.5 | Primary-analysis reference | 4 | 0.217 (-0.423 to 0.857) | 63.80% |
|  | post | core | Sarcopenia-related muscle function domain | 0.7 | Sensitivity analysis: high-correlation assumption | 4 | 0.218 (-0.423 to 0.858) | 63.90% |
|  | post | core | Sarcopenia-related muscle mass/quality domain | 0.3 | Sensitivity analysis: low-correlation assumption | 3 | 0.331 (-0.090 to 0.752) | 0.00% |
|  | post | core | Sarcopenia-related muscle mass/quality domain | 0.5 | Primary-analysis reference | 3 | 0.331 (-0.090 to 0.752) | 0.00% |
|  | post | core | Sarcopenia-related muscle mass/quality domain | 0.7 | Sensitivity analysis: high-correlation assumption | 3 | 0.331 (-0.090 to 0.752) | 0.00% |
|  | post | core | Frailty/overall physical performance domain | 0.3 | Sensitivity analysis: low-correlation assumption | 3 | 0.626 (0.047 to 1.205) | 0.00% |
|  | post | core | Frailty/overall physical performance domain | 0.5 | Primary-analysis reference | 3 | 0.626 (0.047 to 1.205) | 0.00% |
|  | post | core | Frailty/overall physical performance domain | 0.7 | Sensitivity analysis: high-correlation assumption | 3 | 0.626 (0.047 to 1.205) | 0.00% |

Note: This table was used to examine the influence of the rho assumption on the results of the risk-domain composite SMDs. rho = 0.5 was the primary-analysis assumption and served as the primary-analysis reference value in this table; rho = 0.3 and rho = 0.7 represented the low-correlation and high-correlation assumptions in the sensitivity analyses, respectively, and were used to assess the influence of different assumptions regarding correlations among indicators on the direction and magnitude of the pooled effects. SMD > 0 indicates results favoring Tai Chi-based combined interventions. If the direction of the pooled effect and statistical interpretation were broadly consistent under rho = 0.3, 0.5, and 0.7, this suggested that the risk-domain composite SMD results were not sensitive to the correlation assumption. The primary-analysis forest plots are shown in Supplementary Figures S2–S5. This table presents only the rho assumption-related sensitivity analyses for risk-domain composite SMDs and the primary-analysis reference; specific outcome pools, GIV supplementary analyses, and event data were not included in the primary GRADE framework.

**Supplementary Table S6. Sensitivity analysis results for specific outcome pools**

| **Comparison type** | **Data model** | **Analysis level** | **Specific outcome pool** | **k** | **Included studies** | **SMD (95%CI)** | **I^2^** |
| --- | --- | --- | --- | --- | --- | --- | --- |
| Active exercise control | post | core | TUG | 2 | Meng_2022; Sharma_2025 | 0.232 (-1.487 to 1.950) | 0.00% |
| Active exercise control | post | core | Grip strength | 4 | Guo_2024; Meng_2022; Wei_2026; Zhan_2026 | 0.199 (-0.429 to 0.828) | 59.05% |
| Active exercise control | post | core | Muscle mass/quality | 3 | Guo_2024; Wei_2026; Zhan_2026 | 0.143 (-0.188 to 0.474) | 0.00% |
| Non-exercise control | post | core | SPPB | 2 | Liang_2024; Zhan_2026 | 1.458 (-0.728 to 3.644) | 0.00% |
| Non-exercise control | post | core | Sit-to-stand ability | 2 | Chen_2023; Liang_2024 | 1.000 (0.338 to 1.662) | 0.00% |
| Non-exercise control | post | core | Grip strength | 2 | Chen_2023; Zhan_2026 | 1.199 (-2.643 to 5.041) | 32.38% |
| Non-exercise control | post | core | Gait speed/gait ability | 3 | Chen_2023; Liang_2024; Liu_2022 | 1.272 (-0.763 to 3.306) | 83.94% |
| Non-exercise control | post | core | Muscle mass/quality | 2 | Chen_2023; Zhan_2026 | 0.437 (-0.276 to 1.151) | 0.00% |
| Active exercise control | post | core+extended | 6MWT | 2 | Ma_2020_2022; Meng_2022 | 0.528 (-0.401 to 1.457) | 0.00% |
| Active exercise control | post | core+extended | Gait speed/gait ability | 3 | Ma_2020_2022; Meng_2022; Zhang_2021 | 0.933 (0.565 to 1.301) | 0.00% |
| Non-exercise control | post | core+extended | TUG | 2 | Liang_2024; Lin_2015 | 0.702 (-1.145 to 2.550) | 0.00% |
| Non-exercise control | post | core+extended | Lower-limb muscle strength | 3 | Lin_2015; Su_2015; Zhuang_2014 | 0.956 (0.359 to 1.553) | 0.00% |

**Note: This table presents the sensitivity analysis results for specific outcome pools used to explain the risk-domain composite SMD primary analysis. Continuous outcomes were uniformly analyzed using Hedges’ g/SMD, and SMD > 0 indicates results favoring Tai Chi-based combined interventions. Quantitative synthesis was performed only when the same meta-analysis pool included at least 2 independent studies. The primary analysis used a REML random-effects model and Hartung-Knapp adjustment. Specific outcome-pool analyses were used to supplement the interpretation of the clinical sources of risk-domain composite SMDs and did not replace the risk-domain composite SMD primary analysis.**

**Supplementary Table S7. Eligibility assessment for small-study effect/publication bias testing**

| **Analysis scope** | **Number of meta-analysis pools** | **Range of k** | **Status of Egger/funnel plot implementation** | **Description** |
| --- | --- | --- | --- | --- |
| Core primary analyses of risk-domain composite SMDs in the main text | 6 core pooled analyses in the main text | 2–4 | Formal Egger’s test and funnel plot asymmetry assessment were not performed | The core primary-analysis pools corresponding to Table 2 in the main text did not reach the k ≥ 10 threshold. |
| All evaluable pools of risk-domain composite SMDs | 24 analysis pools; 13 pooled with k ≥ 2 and 11 not pooled with k < 2 | 1–8 | Formal Egger’s test and funnel plot asymmetry assessment were not performed | The maximum k was 8 and did not reach the k ≥ 10 threshold; pools with k < 2 were presented only narratively or recorded for eligibility. |
| Specific outcome-pool sensitivity analyses | 23 pooled arm-level pools; 21 unpooled pools with k < 2 | 1–5 | Not performed (all pooled analyses had k < 10) | Only eligibility for testing was assessed, and no statistical inference regarding publication bias was made. |
| GIV supplementary analyses | 7 pooled analyses; 24 unpooled pools with k < 2 | 1–3 | Not performed for any pool | These were supplementary analyses and did not support judgments regarding publication bias. |
| All unpooled pools | 45 pools with k < 2 | 1 | Not applicable | Quantitative synthesis was not performed, and publication bias testing was not conducted. |

**Note: This table explains the eligibility for statistical testing of small-study effects/publication bias and is not intended to demonstrate the absence of publication bias. Because no individual meta-analysis pool in the risk-domain composite SMD analyses, specific outcome-pool sensitivity analyses, or GIV supplementary analyses reached k ≥ 10, formal Egger’s test, funnel plot asymmetry assessment, or trim-and-fill analysis was not performed. Pools with k < 2 were not quantitatively synthesized and were not subjected to publication bias testing.**

**Supplementary Table S8. GRADE robustness checks for risk of bias and imprecision**

| **Check item** | **Summary of results** | **Interpretation** |
| --- | --- | --- |
| RoB 2 exclusion check | Seven bodies of evidence did not include studies with high RoB; four bodies of evidence still had k ≥ 2 after excluding studies with high RoB and therefore theoretically remained eligible for quantitative synthesis; three bodies of evidence had k < 2 after excluding studies with high RoB and could not undergo further quantitative synthesis | The RoB 2 exclusion check showed that some bodies of evidence were influenced by studies at high risk of bias. However, because most meta-analysis pools included only a small number of studies, exclusion of studies at high risk of bias often left too few studies for further quantitative synthesis. Therefore, this check was mainly used to support the interpretation of certainty of evidence and did not replace the primary analysis. |
| Imprecision-threshold scenario analysis | The GRADE ratings of eight bodies of evidence did not change under the current rule, conservative rule, or more lenient threshold rule; the ratings of six bodies of evidence might change under the more lenient rule | Evidence sensitive to imprecision was mainly concentrated in muscle mass/quality and some single muscle function outcomes, for reasons including the small number of studies, 95%CI crossing the null line, or 95%CI crossing the prespecified SMD threshold. |
| Overall robustness judgment | The current GRADE interpretation remained mainly Low or Very low. Because most meta-analysis pools included a limited number of studies, re-pooling after excluding studies with high RoB was not presented as a main result | The overall robustness checks supported interpreting the current evidence as limited or uncertain evidence. This check did not recalculate pooled effect estimates and did not replace the primary GRADE ratings; it was used only to describe the robustness of the evidence interpretation to RoB 2 and imprecision judgments. |

Note: The RoB 2 exclusion check was used to determine whether at least 2 independent studies remained eligible for quantitative synthesis after excluding studies with an overall high RoB. The imprecision-threshold scenario analysis was used to compare whether the certainty of evidence changed under the current rule, conservative rule, and more lenient threshold rule. These robustness checks did not recalculate pooled effect estimates and did not replace the primary analysis or the primary GRADE ratings.

**Supplementary Table S9. Cluster adjustment and ICC assumptions for cRCTs**

| **Study** | **Data use** | **Clustering unit** | **Number of clusters** | **Original n (I/C)** | **Mean cluster size (I/C)** | **ICC** | **Design effect (I/C)** | **n_eff (I/C)** | **ICC source or assumption basis** | **Correction method** |
| --- | --- | --- | --- | --- | --- | --- | --- | --- | --- | --- |
| Liu_2022 | frailty phenotype score; main/core continuous | nursing home | 8; 4 per group | 67 / 68 | 16.75 / 17.00 | 0.051 | 1.8032 / 1.8160 | 37.1551 / 37.4449 | Kendhapedi_2019_Fried_frailty_phenotype; borrowed ICC related to the Fried frailty phenotype | ESS+ICC; for continuous outcomes, means and SDs were unchanged, and only the sample size was adjusted |
| Liu_2022 | stride velocity; main/core continuous | nursing home | 8; 4 per group | 67 / 68 | 16.75 / 17.00 | 0.1 | 2.5750 / 2.6000 | 26.0194 / 26.1538 | Perera_2018_gait_speed; borrowed gait speed ICC in older adults | ESS+ICC; for continuous outcomes, means and SDs were unchanged, and only the sample size was adjusted |
| Liu_2022 | step length, cadence; secondary gait continuous | nursing home | 8; 4 per group | 67 / 68 | 16.75 / 17.00 | 0.1 | 2.5750 / 2.6000 | 26.0194 / 26.1538 | Perera_2018_gait_speed_borrowed_for_gait_parameters; both were gait parameters, and gait speed ICC was borrowed | ESS+ICC; for continuous outcomes, means and SDs were unchanged, and only the sample size was adjusted |
| Liu_2022 | stride time; secondary gait continuous | nursing home | 8; 4 per group | 67 / 68 | 16.75 / 17.00 | 0.06 | 1.9450 / 1.9600 | 34.4473 / 34.6939 | Perera_2018_stance_time_variability; borrowed ICC related to temporal gait parameters | ESS+ICC; for continuous outcomes, means and SDs were unchanged, and only the sample size was adjusted |
| Lin_2015 | 30s arm curl, 30sCST, 8-foot up-and-go, left/right knee extension; main/core continuous | senior day care centre | 6; 3 per group | 48 / 47 | 16.00 / 15.67 | 0.05 | 1.7500 / 1.7335 | 27.4286 / 27.1128 | functional_fitness_older_adult_cluster_trial_or_Perera_SPPB; borrowed ICC related to physical fitness/physical performance in older adults | ESS+ICC; for continuous outcomes, means and SDs were unchanged, and only the sample size was adjusted |
| Lin_2015 | 6MWT; main/core continuous | senior day care centre | 6; 3 per group | 48 / 47 | 16.00 / 15.67 | 0.09 | 2.3500 / 2.3203 | 20.4255 / 20.2560 | Perera_2018_6MWD; borrowed 6-minute walk distance ICC | ESS+ICC; for continuous outcomes, means and SDs were unchanged, and only the sample size was adjusted |
| Lin_2015 | back scratch, chair sit-and-reach, upper/hip strength MMT, and other supplementary continuous outcomes | senior day care centre | 6; 3 per group | 48 / 47 | 16.00 / 15.67 | 0.05 | 1.7500 / 1.7335 | 27.4286 / 27.1128 | functional_fitness_older_adult_cluster_trial_or_Perera_SPPB; outcomes also related to physical fitness or physical performance in older adults | ESS+ICC; for continuous outcomes, means and SDs were unchanged, and only the sample size was adjusted |
| Lin_2015 | falls; secondary dichotomous event | senior day care centre | 6; 3 per group | 48 / 47 | 16.00 / 15.67 | 0.071 | 2.0650 / 2.0416 | 23.2446 / 23.0215 | falls_Cameron_2018_Dyer_2004_number_residents_falling; borrowed ICC related to fall events | ESS+ICC; both the number of participants and the number of events were adjusted by the design effect |
| Woo_2024 | not included in the ESS+ICC-corrected main effect-size table | cluster / quasi-cluster allocation | NR | 34 for comparison-only data | NR | NA | NA | NA | The original data were comparison-only or had insufficient cluster correction information; arm-level ESS+ICC input could not be generated | ESS+ICC replacement was not performed; not used as current main-model cluster-adjusted arm-level data |

Note: I indicates intervention group; C, control group; ICC, intracluster correlation coefficient; ESS, effective sample size; DE, design effect; n_eff, effective sample size adjusted by the design effect. The design effect was calculated as DE = 1 + (M − 1) × ICC, where M is the mean cluster size. For ESS correction of continuous outcomes, the means and SDs were kept unchanged, and only the original sample size was replaced with n_eff; for dichotomous event data, both the number of participants and the number of events were adjusted by DE. n_eff_unrounded was used for R/meta calculations, and n_eff_rounded_for_RevMan was used only for software display requiring integer sample sizes. Outcomes with borrowed ICCs were treated as sensitivity and transparency considerations in interpretation and were not used as sources of high-certainty evidence.

**Supplementary Figure S1. RCT risk-of-bias plot**

Note: This figure shows the summary results of the RoB 2 risk-of-bias assessment for the included individually randomized controlled trials. The assessment domains included bias arising from the randomization process, bias due to deviations from intended interventions, bias due to missing outcome data, bias in measurement of the outcome, bias in selection of the reported result, and overall risk of bias. Green indicates low risk of bias, yellow indicates some concerns, and red indicates high risk of bias. Cluster-randomized trials were assessed using the cluster-RoB 2 framework, and the main judgments are summarized in the risk-of-bias section of the main text; because of limitations in the official summary-plot format, cRCT results are not repeatedly presented as a separate summary plot.

**Supplementary Figure S2. Forest plots of risk-domain composite SMDs at the core level for non-exercise control**

Supplementary Figure S2A: Sarcopenia-related muscle function domain

Supplementary Figure S2B: Sarcopenia-related muscle mass/quality domain

Supplementary Figure S2C: Frailty/overall physical performance domain

**Note: This figure shows the primary analysis results for risk-domain composite SMDs at the core level under non-exercise control. The direction of all effect sizes was harmonized so that SMD > 0 indicates results favoring Tai Chi-based combined interventions. Blue squares indicate the study-level composite SMDs of individual studies, and horizontal lines indicate 95%CIs; blue diamonds indicate pooled effects from the random-effects model, and red horizontal lines indicate prediction intervals. The model used REML to estimate τ² and used the Hartung-Knapp method to calculate pooled effects and 95%CIs. The core-level results served as the main basis for interpretation of the primary analysis in the main text.**

**Supplementary Figure S3. Forest plots of risk-domain composite SMDs at the core+extended level for non-exercise control**

Supplementary Figure S3A: Sarcopenia-related muscle function domain

Supplementary Figure S3B: Sarcopenia-related muscle mass/quality domain

Supplementary Figure S3C: Frailty/overall physical performance domain

**Note: This figure shows the supplementary analysis results for risk-domain composite SMDs at the core+extended level under non-exercise control. The core+extended level included extended risk populations in addition to the core-level population and was used to examine whether the direction of results remained consistent after broadening the population scope. Results at this level were used for robustness and generalizability judgments and did not take priority over the core-level primary analysis results. SMD > 0 indicates results favoring Tai Chi-based combined interventions. Blue squares indicate effect sizes from individual studies, blue diamonds indicate pooled effects from the random-effects model, and red horizontal lines indicate prediction intervals.**

**Supplementary Figure S4. Forest plots of risk-domain composite SMDs at the core level for active exercise control**

Supplementary Figure S4A: Sarcopenia-related muscle function domain

Supplementary Figure S4B: Sarcopenia-related muscle mass/quality domain

Supplementary Figure S4C: Frailty/overall physical performance domain

**Note: This figure shows the primary analysis results for risk-domain composite SMDs at the core level under active exercise control. Active exercise control included non-Tai Chi active exercise, usual exercise training, or other control conditions with clear exercise stimuli, and was used to evaluate whether Tai Chi-based combined interventions had additional benefits compared with other active exercise programs. SMD > 0 indicates results favoring Tai Chi-based combined interventions. Blue squares indicate effect sizes from individual studies, blue diamonds indicate pooled effects from the random-effects model, and red horizontal lines indicate prediction intervals. The core-level results served as the main basis for interpreting comparisons with active exercise control in the main text.**

**Supplementary Figure S5. Forest plots of risk-domain composite SMDs at the core+extended level for active exercise control**

Supplementary Figure S5A: Fall-risk/balance-mobility domain

Supplementary Figure S5B: Sarcopenia-related muscle function domain

Supplementary Figure S5C: Sarcopenia-related muscle mass/quality domain

Supplementary Figure S5D: Frailty/overall physical performance domain

**Note: This figure shows the supplementary analysis results for risk-domain composite SMDs at the core+extended level under active exercise control. The core+extended level was used to examine whether the direction of effects remained consistent after including extended risk populations. The fall-risk/balance-mobility domain had an insufficient number of combinable studies at the core level and was therefore presented only as a supplementary result at the core+extended level. The results of this figure set were not used as a basis for conclusions that took priority over the core-level primary analysis. SMD > 0 indicates results favoring Tai Chi-based combined interventions. Blue squares indicate effect sizes from individual studies, blue diamonds indicate pooled effects from the random-effects model, and red horizontal lines indicate prediction intervals.**

**Supplementary Figure S6. Forest plot of the age-boundary sensitivity analysis excluding Sharma et al.**

**Note: This figure shows the active-exercise-control, post-intervention, core-layer frailty/overall physical performance composite after excluding Sharma et al. The same REML random-effects model with Hartung-Knapp adjustment was used. SMD > 0 indicates results favoring Tai Chi-based combined interventions. The analysis retained Meng_2022 and Zhan_2026 (k = 2) and yielded SMD = 0.738 (95%CI: −2.639 to 4.114; I² = 49.15%). The direction remained favorable, but the confidence interval widened markedly. This analysis assessed the influence of retaining an age-boundary trial and did not estimate age-specific treatment effects.**
